# The effectiveness of interventions to disseminate the results of non-commercial randomised clinical trials to healthcare professionals: a systematic review

**DOI:** 10.1186/s13012-023-01332-w

**Published:** 2024-02-01

**Authors:** Annabelle South, Julia V. Bailey, Mahesh K. B. Parmar, Claire L. Vale

**Affiliations:** 1 MRC Clinical Trials Unit at UCL, Institute of Clinical Trials and Methodology, UCL, 90 High Holborn, London, WC1V 6LJ UK; 2 Research Department of Primary Care and Population Health, UCL, London, UK

**Keywords:** Communication, Dissemination, Clinical studies, Health professionals, Policymakers, Knowledge broker, Outreach, Academic detailing

## Abstract

**Background:**

It is unclear how to disseminate the results of randomised controlled trials effectively to health professionals and policymakers to improve treatment, care or prevention through changing policy and practice. This systematic review examined the effectiveness of different methods of dissemination of clinical research results to professional audiences.

**Methods:**

We systematically reviewed the published and grey literature from 2000 to 2022 for studies assessing different approaches for disseminating clinical study results to professional audiences (health professionals, policymakers and guideline developers). Two reviewers assessed potentially relevant full texts for inclusion. We grouped studies by intervention type, synthesising findings using effect direction plots. Outcomes were grouped into out-takes (e.g. awareness, knowledge, understanding), outcomes (e.g. attitude changes) and impact (changes in policy/practice). The quality of evidence was assessed using GRADE.

**Results:**

Our search identified 13,264 unique records, of which 416 full texts were assessed for eligibility. Of 60 studies that were identified as eligible for inclusion, 20 evaluated the effectiveness of interventions to disseminate clinical research results (13 RCTs, 2 observational studies, 3 pre- and post-intervention surveys and 2 cross-sectional surveys). Studies were grouped by intervention: 7 studies that involved face-to-face meetings between the target audience and trained educators were classified as ‘outreach interventions’; 5 studies that provided a summary format for systematic review findings (e.g. summary of findings tables) were grouped together. There was high certainty evidence of a small beneficial impact of outreach interventions on health and moderate certainty evidence of impact on practice (mostly prescribing). There was no evidence of impact on policy and very low certainty around benefits on outcomes and out-takes. We found no consistent benefits of summary formats for systematic review results on outcomes or out-takes (moderate quality evidence). Other interventions with less evidence are reported in the Additional Materials.

**Conclusions:**

Outreach interventions to disseminate clinical research results can lead to changes in practice and improvements in health. However, these interventions can be resource-intensive. Investment is vital to identify and implement effective and cost-effective ways to disseminate results, so that the potential benefits of trials to patients can be realised.

**Trial registration:**

International Prospective Register of Systematic Reviews (PROSPERO), CRD42019137364.

**Supplementary Information:**

The online version contains supplementary material available at 10.1186/s13012-023-01332-w.

Contributions to the literature

- This paper reports the results of a comprehensive systematic review evaluating the effectiveness of approaches to disseminating clinical study results to professional audiences including health professionals and policymakers.
- There was good evidence of effectiveness for outreach approaches (meetings with health professionals), but these can be resource-intensive.
- There is little high-quality evidence available for other approaches to disseminating results, leading to uncertainty around the effectiveness of approaches such as knowledge broker services and researchers repackaging study results.
- More research is needed to guide researchers on how best to disseminate and communicate results to professional audiences in a cost-effective manner

## Background

Phase III randomised controlled trials are often costly and can take years to carry out [1]. Trials may involve hundreds or thousands of participants, cared for by health professionals at many sites. They aim not just to increase the sum of human knowledge, but also to improve treatment, care or prevention for future patients. However, the results of clinical trials and systematic reviews may take years to change policy and practice [2, 3]. To achieve changes in policy and practice, the results, in the context of the global evidence base [4], need to be disseminated and communicated effectively to a variety of audiences [5], as a key part of the knowledge translation process [6–13].

Evidence on how best to disseminate and communicate trial results to different audiences is sparse [5]; therefore, time and resources may be wasted on ineffective activities, while effective approaches are not widely used. Passive approaches to dissemination are ineffective [14]. A review by the Agency for Healthcare Research and Quality (AHRQ) [5] evaluated communication and dissemination strategies to facilitate the use of health-related evidence, reporting the comparative effectiveness of the dissemination strategies to promote the use of healthcare evidence. They defined dissemination as ‘the active and targeted distribution of information or interventions via determined channels using planned strategies to a specific public health or clinical practice audience’ [5]. They focused on dissemination strategies to increase reach of information, motivation to use and apply evidence, and ability to use and apply evidence or used a multicomponent approach but found that evidence was poor, inconsistent or not statistically significant for most of the comparisons they evaluated. The most successful strategy they identified was the use of a multicomponent dissemination approach addressing a combination of reach, ability or motivation for clinicians when trying to change their behaviours. They also looked at communication strategies focusing on ‘making evidence interpretable, persuasive and actionable’ [5], such as tailoring information to individuals, targeting information to specific sub-groups, using narratives and framing the message. We view the concepts of dissemination and communication as distinct and complimentary—different dissemination approaches to distributing information or interventions can be combined with different communication approaches used within the information or intervention that is being disseminated.

Professional audiences such as health professionals and policymakers are not the only important audiences for the results of clinical studies. There is a growing evidence base on how to share the results of clinical studies to participants [15–18]. However, there are likely to be major differences in the most appropriate and effective methods for these different audiences, as not only will the content of dissemination and communication interventions need to be different, but also the aim of disseminating results to the different audiences may also be different (for participants it is about fulfilling an ethical duty to inform, whereas for health professionals and policymakers the focus may be more on changing behaviour).

Communication and dissemination interventions may be associated with a range of potential outcomes, from changes in knowledge through to changes in policy or practice (which may require additional interventions or supportive contexts to achieve). The International Association for the Measurement and Evaluation of Communication Framework categorises the potential outcomes from communication into outputs (the content, materials and activities shared with target audiences), out-takes (what the target audience takes out of the communication, and how they react to it), outcomes (the effects of the communication on the target audience) and impact (the results that are caused, in full or in part, by the communication) [19]. While it is unrealistic to expect to see ‘impact’-type outcomes from the dissemination and communication of the results of all trials and systematic reviews, there are circumstances in which the results of clinical studies have rapidly led to changes in policy and practice, once disseminated. For example, the results of the START trial, presented and published in 2015, led to immediate changes in HIV treatment guidelines around the world, with World Health Organization data from 2016 showing that the proportion of low- and middle-income countries applying a ‘treat all’ policy for antiretroviral therapy had doubled since 2015 [20]. Our new systematic review aimed to examine the effectiveness of different methods of dissemination and communication of clinical research results to professional audiences (health professionals, policymakers, clinical guideline developers and healthcare commissioners). It builds on the AHRQ review by including non-experimental studies alongside trials of dissemination approaches and by including comparative evidence that has been generated since the AHRQ review to evaluate the effectiveness of different approaches to disseminating and communicating the results of clinical research to professional audiences.

The question of this review was: How effective are different approaches to disseminating and communicating the results of clinical research to professional audiences? The PICO for this review can be found in Table 1. We aimed to learn lessons that may be applicable to phase III non-commercial clinical trials, as well as to clinical trials more broadly, to inform the practice of clinical trials units, systematic reviewers and others who are interested in effectively disseminating and communicating clinical research results. We hope this will inform the dissemination approaches used by researchers, improving the translation of research into out-takes and outcomes among professional audiences, ultimately leading to impact on policy and practice, improving health for patients.

**Table 1:** PICO for the review

Population: professional audiences	The population for our review was health professionals, policymakers, guideline developers and healthcare commissioners, not restricted by age, sex, location or other demographic factors
Intervention: interventions to communicate or disseminate the results of clinical studies	We were interested in interventions aiming to disseminate or communicate the overall results of clinical studies to the population described above. We were primarily interested in interventions sharing the results of phase III non-commercial clinical trials but also explored the literature on disseminating the results of other clinical research study designs that are likely to generate evidence with direct implications for policy and practice (for example, systematic reviews and cohort studies). We did not pre-specify which interventions (dissemination methods) we were interested in, as we wished to be inclusive of all strategies that had been examined in the literature
Comparator/study design: no comparator specified	We did not specify a particular comparator or study design
Outcomes: changes in policy and clinical practice	We explored a range of outcomes, covering the out-takes, outcomes and impact dimensions of the International Association for the Measurement and Evaluation of Communication Framework [4]. Our co-primary outcomes were changes in the recommendations made in clinical policy documents and clinical guidelines published by professional bodies or government agencies and changes in clinical practice

## Methods

The full protocol for our systematic review has been published [18] and is available in PROSPERO (CRD42019137364). This paper reports results relating to the effectiveness of interventions for disseminating results to professional audiences; results relating to other questions from our review will be reported separately. While our search strategy included both dissemination and communication concepts, we only found studies that assessed the effectiveness of dissemination strategies, not communication strategies, so this paper focuses on dissemination.

### Searches

Our search strategy was developed through extensive testing and refinement, with input from an information scientist. We searched Embase, MEDLINE, PsycINFO, ASSIA and the Cochrane Database of Systematic Reviews and ProQuest Dissertations & Theses Global, from 01/01/2000 to 22/04/2022. We also searched the proceedings from relevant conferences including the Society for Clinical Trials, the International Clinical Trials Methodology Conference and the Cochrane Colloquium (the full list of information sources is in Additional file 1, section A1.2). Search terms covering the concepts of dissemination/communication, audience and clinical research were combined using adjacency rules (where available in the database interface) to limit results to articles reporting relevant audience, dissemination/communication and clinical research terms (list available in Additional file 1, section A1.1). The title, abstract and subject heading fields were searched. For databases where we were able to use adjacency rules, this approach meant we were unable to use subject headings; however, our testing of the search strategy revealed that searches using the adjacency approach were more sensitive and specific than those using subject headers. Where the adjacency function was not available, we used MeSH terms to narrow down our results. We developed and pilot tested the full search strategy in the Embase database, using the Ovid interface, until we arrived at a strategy that was sufficiently sensitive and specific. Search strategies including subject headings and syntax were all adapted to the individual databases. Some of the grey literature sources had less sophisticated search functions. For these, we used broad search terms from our list and hand searched the results to identify those that were potentially relevant. All searches were limited to English. No study design limits were imposed on the search. We also hand searched the references of included studies and asked other researchers for references that we should be aware of. Full details are given in our published protocol [18].

### Study inclusion and exclusion criteria

We defined professional audiences as follows:

- Health professionals, including individual practitioners, organisations (e.g. hospitals or medical schools) and professional associations/societies
- Policymakers
- Clinical guideline developers
- Healthcare commissioners

We did not restrict the population by age, sex, location or other demographic factors.

We were interested in any approaches for disseminating or communicating the results of clinical studies carried out on humans that have implications for health policy or practice to any of the populations specified above. We did not restrict the search to studies with a particular comparator.

We excluded articles that were:

- Published prior to 1 January 2000
- Not about a clinical study (e.g. articles about the communication of clinical guidelines or decision aids, or not about specific clinical studies—this was determined through checking the references cited in the description of what was being communicated)
- Not about dissemination/communication (e.g. articles reporting results of a clinical study, rather than reporting how those results were disseminated/communicated)
- Not about dissemination/communication of study results (articles about dissemination/communication of other aspects of studies, rather than results)
- Not assessing the effectiveness of dissemination/communication approaches (e.g. studies exploring user feedback on dissemination tools without seeking to measure outcomes from those tools or reporting solely qualitative results (these will be reported in the second phase of our review))
- Focused solely on disseminating/communicating results to lay audiences such as patients and the public, rather than professionals (these will be included in the third phase of our review)
- Commentaries, editorials, letters, protocols and systematic reviews (although the references of these were reviewed for relevant studies)
- Not written in English

We used the International Association for the Measurement and Evaluation of Communication Framework [19] to categorise outcomes of interest as *out-takes* (defined as what the audience take from the dissemination/communication, including awareness, knowledge and understanding), *outcomes* (defined as attitudes towards the health intervention, including interest, support and intention to adopt the findings) and *impact* (defined as changes in policy or practice, including prescribing). Table 2 shows the out-takes, outcomes and impacts of interest for the review. The type of outcomes reported were not an inclusion or exclusion criterion. The length of follow-up or point at which outcomes were measured was not an eligibility criterion.

**Table 2:** Outcomes of interest

Out-takes	Outcomes	Impact
Awareness Knowledge Understanding/comprehension/clarity	Attitude to the results (e.g. interest, consideration, support, intention to adopt findings)	**Changes in practice** (including changes in prescribing patterns) **Changes in policy** **Changes in guidelines**

AS assessed the eligibility of titles and abstracts identified from electronic searches against the eligibility criteria of the review, discarding only those that are duplicates or clearly irrelevant. AS and CV then independently assessed full-text copies of all potentially eligible articles for inclusion, meeting regularly to ensure the eligibility criteria are being applied consistently and resolve disagreements. Where there was insufficient information in the publication to assess its eligibility, AS contacted the authors to request additional information.

### Data extraction strategy

We extracted data relating to the study design, setting, results to be disseminated, dissemination/communication approach(es) used, target audience and outcomes using standard electronic forms based on the Cochrane Consumers and Communication Group’s data extraction template [21], adapted to fit the needs of this review. The forms were piloted in the first five studies. Our extraction form can be found as an additional file with our published protocol [18]. Data were extracted by AS with queries resolved through discussion with CV. Where data items were missing for a study, we attempted to contact the authors to obtain it. Where there were multiple reports from the same study, articles were grouped together and the most up-to-date data used for each outcome.

### Study quality assessment

We assessed study quality using an appropriate, recognised tool for each eligible study. We used the Cochrane ‘RoB 2.0’ tool [22] for individually randomised controlled trials or cluster randomised and the ROBINS-I tool [23] for cohort studies and case–control or the AXIS tool [24] for cross-sectional studies. We categorised studies as low risk of bias, some concerns or high risk of bias and included this information in the effect direction plots at the data synthesis stage.

We graded the overall quality of the synthesised quantitative evidence for each outcome separately, following the GRADE guidelines, as high, moderate, low or very low, taking into account the risk of bias, effect size, consistency of results, directness of evidence, precision and risk of publication bias [25].

### Data synthesis and presentation

Once data had been extracted, but prior to analysis, we looked at the included studies to see how best to group them (based on interventions, populations or outcomes). As the populations and outcomes varied substantially between the studies, we decided to group them into four broad categories of interventions for synthesis and presentation. Within the intervention groups, there were similarities in the types of outcomes reported (e.g. out-takes, outcomes and impacts), but the details of the outcomes varied considerably.

We summarised the characteristics and findings of the included studies, grouped by intervention type in text and tables. Findings across studies were synthesised using effect direction plots [26, 27], combining findings from similar outcomes within each study into a single outcome domain (based on the AMEC integrated evaluation framework [19]). The impact was split into four sub-domains: impact on prescribing, impact on other practice outcomes (such as the use of non-pharmaceutical approaches, composition of the team involved in treating patients or use of shared decision-making), impact on policy and impact on health outcomes. We derived an overall direction of effect for each outcome domain (or sub-domain) reported in each study using a 70% threshold as described by Thomson et al. [27] and the algorithm as follows:

- Benefit: in order to be classed as beneficial, the direction of effect of ≥ 70% of outcomes within the sub-domain/domain within a study must benefit from the intervention.
- Detriment: in order to be classed as detrimental, the direction of effect of ≥ 70% of outcomes within the sub-domain/domain within a study must be detriment from the intervention.
- No change/inconsistent: if the direction of effect of < 70% of outcomes within the sub-domain/domain is the same, we class the intervention as having no change/inconsistent findings for that domain/sub-domain in that study.

The *p*-value of each individual outcome was not considered when classifying the effect direction [28]. Meta-analysis was not carried out as the available data did not meet the pre-specified criteria (i.e. low risk of bias in the included studies, consistent outcomes between studies, low publication bias, a high number of included studies and low heterogeneity [29, 30]). Instead, we used a sign test [26] to estimate the probability of a given number of positive and negative results being observed if the null hypothesis was true. To carry out the test, we counted the number of benefit and detriment arrows for each outcome domain (excluding studies with inconsistent effect direction for that domain) and used the Microsoft Excel BINOM.DIST function to calculate the *p*-value for each domain. Due to insufficient evidence, we were unable to explore whether the effectiveness of dissemination approaches varies by the target audience, disease or geographical location.

## Results

### Search results

**Fig. 1:**
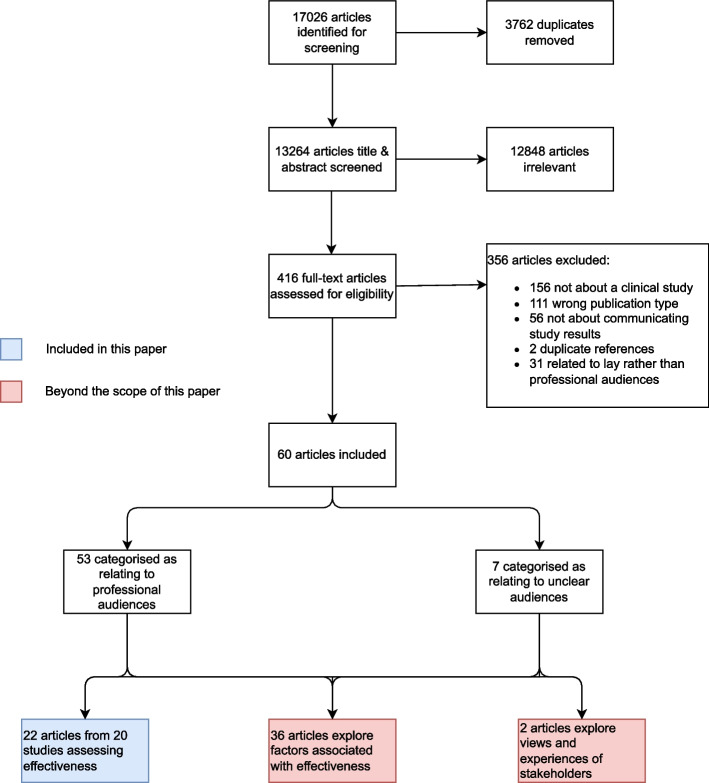
PRISMA flow diagram

We identified 17,026 articles for screening, of which 3,762 were removed as duplicates. A total of 13,264 articles were screened by title and abstract, of which 12,848 were deemed irrelevant (most reported results of clinical studies, rather than the communication of clinical study results). A total of 416 full-text articles were assessed for eligibility, of which 356 were excluded (156 were not about clinical studies; 111 were ineligible publication types; 56 were not about communicating study results; 2 were duplicates; 31 related to communicating results to lay rather than professional audiences). A total of 60 articles were deemed eligible for inclusion in the review, of which 53 were categorised as being about professional audiences, and 7 were categorised as being unclear about which audience they referred to. A total of 22 articles from 20 studies assessed the effectiveness of dissemination interventions. No studies evaluated the effectiveness of communication strategies. Figure 1 shows the PRISMA flow diagram for our review. For ease of analysis and interpretation, we grouped studies into 4 categories, according to the nature of the intervention: (1) outreach interventions, (2) summary formats for systematic reviews, (3) knowledge broker interventions and (4) researchers repackaging results. Of the 4 studies that relate to knowledge broker interventions, only 1 was considered at low risk of bias, and of the 5 studies that report researchers repackaging results, none was at low risk of bias, giving us very little evidence upon which to draw conclusions around the effectiveness of these approaches. Therefore, the remainder of this report focuses on the first two intervention types (outreach interventions and summary formats for systematic reviews). The results relating to knowledge brokers and researchers repackaging results are presented in Additional files 2 and 3, respectively.

### Outreach interventions

Outreach interventions: these all involved face-to-face meetings between the target audience (healthcare practitioners) and trained educators, some with additional components. This included ‘academic detailing’ interventions (using the outreach strategies employed by pharmaceutical companies for disseminating unbiased evidence and recommendations on a topic) [31]. Details of the implementation strategies that were part of these interventions can be found in Additional file 4: Table A4.1.

#### Included studies

Seven eligible studies assessed outreach interventions (Table 3 and Additional file 4: Table A4.1). The interventions assessed often included several implementation strategies, which we classified in Additional file 4: Table A4.1 using the ERIC classification [32]. Interventions included meetings with individuals [33, 34] or groups of health professionals from the same health facility [35–37] or local area [38]. In some studies, these meetings were supplemented with educational materials [35–38]. Some interventions focused on presenting the evidence [33, 36, 38], whereas other, more intensive interventions, also sought to address the change process needed to implement the recommendations [35, 37]. Interventions were delivered by clinicians [33, 35, 38], pharmacists [33, 36] and research nurses [37]. Some interventions involved one-off outreach to target audiences [33, 36, 38], whereas others, which focused on the change process in addition to the evidence, involved more sustained contact with the target audience over a period of time [35, 37]. The approach was tailored to the specific audience in most of the interventions included in this category [33, 35, 37, 38].

**Table 3:** Studies relating to outreach interventions (ordered by study design and risk of bias)

Study ID	Design	Setting	Length of follow-up	Intervention groups	Audience	Goal(s) of dissemination	Risk of bias assessment
Skoglund 2013 [33]	Cluster randomised controlled trial (evidence-based drug information vs evidence-based drug information + motivational interviewing)	Primary care health centres in Sweden	6 months follow-up post-intervention	Evidence-based drug information + motivational interviewing (408 GPs) vs evidence-based drug information alone (control) (583 GPs)	Practitioners: primary care GPs	Reach, motivation	Low risk of bias
Ludden 2018 [37]	Cluster randomised controlled trial (facilitator-led dissemination vs traditional dissemination vs no intervention)	Primary care practices in North Carolina, USA	3 months follow-up	Facilitator-led approach to dissemination (10 clusters) vs traditional dissemination (10 clusters) vs control (no formal dissemination) (10 clusters)	Primary care providers	Reach, ability to implement change	Low risk of bias
Acolet 2011 [35]	Cluster randomised controlled trial (control arm vs active dissemination)	180 neonatal units in the UK	6 months follow-up	Active dissemination: dissemination—as below + regional champions, workshops, benchmarking and email and telephone support (87 units) vs control arm: report, recommendations and slide set sent to units (93 units)	Staff at neonatal units	Reach, ability to implement change	Some concerns due to missing outcome data and discrepancies in the number of units reported as randomised to intervention/control in protocol vs results paper
Bernal-Delgado 2002 [36]	Pragmatic, simple blind trial, with random assignation to one of three groups	Primary care practices in Spain	6 months post-intervention	Experimental group (participating in an evidence-based educational outreach visit) (48 GPs) vs placebo group (participating in a conventional educational session), and control group (with no intervention) (56 GPs)	GPs in Spain	Reach	Some concerns due to a lack of information about missing data and deviations from intended interventions
Stafford 2010 [38]	Observational study using survey data and pharmacy data	Primary care practices in the USA	9-months pre-intervention to 9 months post-intervention	Comparing intensity (number of physicians contacted by investigator-educators per 100,000 population members 50 years or older) of dissemination efforts (academic detailing) with outcomes from geographic areas	Community physicians in the USA	Reach, motivation, ability to implement change	Some concerns around potential for confounding^a^
Bartholomew, 2009 [39]	Process evaluation, including pre and post-intervention questionnaires	Primary care practices in the USA	12 months	Academic detailing	Internal medicine and family practitioners	Reach, motivation, ability to implement change	Some concerns around selection bias, lack of information on response rate and statistical methods^a^
Majumdar 2015 [34]	Observational study looking at the prescription of ramipril in two countries, one with no promotion (USA) and one with promotion (Canada)	Pharmacies in Canada and the USA	4 years of follow-up	Promotional activity undertaken by pharmaceutical companies (Canada) (4800 pharmacies) vs no promotional activity undertaken by pharmaceutical companies (USA) (51,355 pharmacies)	Cardiologists	Reach, ?	Serious risk of bias due to the potential for confounding^b^

^a^Risk of bias for this study assessed using AXIS

^b^Risk of bias for this study assessed using ROBINS-1

All the studies in this intervention category had health professionals as their audience. Seven studies targeted clinicians [33–39], with one targeting pharmacists [33]. Five of the studies focused on primary care clinicians [33, 36–39] while two focused on secondary or tertiary care clinicians [34, 35]. Studies disseminated information about cardiovascular disease [33, 34, 38, 39], neonatal care [35], arthrosis [36] and asthma [37]. All studies were carried out in high-income countries, in either Europe [33, 35, 36] or North America [34, 37–39].

Five of the studies used multicomponent strategies, targeting improving reach, alongside the ability to use and apply the evidence, and/or motivation to use and apply the evidence [33, 35, 37–39]. All the studies that evaluated outreach interventions measured the impact of those interventions. The most common type of impact assessed was change in clinical practice, specifically around prescriptions [33, 34, 36–38]. We have categorised changes in prescribing patterns as an impact on practice rather than an impact on health outcomes, as changes in practice may not necessarily result in changes in health outcomes for patients (for example, if the change in prescribing patterns is based on the results of a non-inferiority trial). Acolet 2011 assessed the changes in hospital/unit policies and strategies, changes in practice and changes in patient outcomes [35]. Ludden 2018 also assessed the impact of the intervention on patient health outcomes [37]. They also evaluated the changes in patient perceptions of shared decision-making.

Only one study of outreach interventions reported outcomes that fit in the ‘Outcome’ or ‘Out-take’ columns of the AMEC framework. They assessed reported intention to change practice following receipt of an outreach visit (an ‘outcome’) and expectations and beliefs around the treatment approach the intervention focused on (‘out-takes’) [39].

#### Study quality assessment

The quality of evidence around the outreach interventions varied between the studies. Four studies used a randomised controlled trial design [33, 35–37], while the remaining two studies were observational in design [34, 38, 39]. Two of the randomised controlled trials [33, 37] were judged to be at low risk of bias. Additional file 5: Table A5.1 shows the GRADE rating of certainty of evidence for studies in this category.

#### Evidence of effectiveness

Table 4 summarises the effect directions for the different outcome types measured in the studies, and Table 5 shows the summary of findings for outreach interventions. Additional file 6: Table A6.1 summarises the results of the studies assessing the effectiveness of outreach interventions.

**Table 4:**
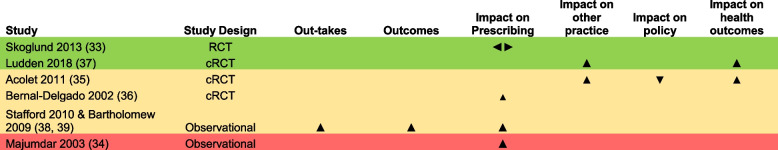
Effect direction plot for outreach interventions (ordered by study design and risk of bias) [33–39]

Study design: *RCT* randomised controlled trial, *cRCT* cluster randomised trial

Effect direction: upward arrow ▲ = positive health impact; downward arrow ▼ = negative health impact; sideways arrows ◄► = no change/mixed effects/conflicting findings

Sample size: final sample size (individuals) in the intervention group: large arrow  > 300; medium arrow  50–300; small arrow  < 50

Study quality: denoted by row colour: green = low risk of bias; amber = some concerns; red = high risk of bias

**Table 5:** Summary of findings for outreach interventions on outcomes assessed

Outcome assessed	Effect	Number of participants (studies)	Certainty in the evidence^a^
Impact on practice (prescription and other practice outcomes)	Most studies showed beneficial changes in practice^b^	The individually randomised study had 991 participants [33]. The cluster-randomised trials had 234 clusters in total [35–37]. Two studies were very large observational studies collecting data from thousands of pharmacies [34, 38] (6 studies)	Moderate certainty^c^ ⊕ ⊕ ⊕ ◯
Impact on policy	No benefit from outreach interventions on policy and some suggestion of detriment (which is likely explained by residual confounding)	One cRCT with 180 clusters [35] (1 study)	Moderate certainty^d^ ⊕ ⊕ ⊕ ◯
Impact on health outcomes	Both studies reported small benefits in most health outcomes measured (with one outcome showing a small detriment)	One cRCT with 30 clusters (6274 patients) [37] and the other with 180 clusters (355 patients) [35] (2 studies)	High certainty ⊕ ⊕ ⊕ ⊕
Outcomes	The only study looking at this reported consistent benefit	One observational study with data from 2640 respondents [39] (1 study)	Very low certainty^e^ O OOO
Out-takes	The only study looking at this reported consistent benefit	One observational study with data from 2640 respondents [39] (1 study)	Very low certainty^e^ O OOO

^a^Commonly used symbols to describe certainty in evidence in evidence profiles: high certainty (⊕ ⊕ ⊕ ⊕), moderate certainty (⊕ ⊕ ⊕ O), low certainty (⊕ ⊕ OO) and very low certainty (⊕ OOO)

^b^One study at low risk of bias reported inconsistent outcomes. The other studies all reported benefits

^c^Downgraded by one level for inconsistency

^d^Downgraded by one level for risk of bias concerns, one level for imprecision and upgraded by one level for plausible residual confounding

^e^Downgraded by one level due to risk of bias concerns around potential selection bias, lack of information on response rate and statistical methods

##### Impact

Overall, the studies suggest that outreach interventions may have an impact on practice (combining prescribing behaviour and other practice outcomes) (sign test *p*-value for impact on practice sub-domains 0.031) which could potentially impact on health outcomes (Tables 4 and 5). However, there is no evidence that these interventions can change policy. Combining results from all the impact sub-domains, the sign test *p*-value was 0.031. The results suggest that overall, outreach interventions have a benefit, but there is not yet enough evidence to be confident this is the case in any single impact sub-domain (prescribing, other practice, policy or health outcomes).

##### Impact on practice

There is moderate certainty evidence that outreach interventions may lead to an impact on practice (Tables 4 and 5). The *p*-value for the impact on the practice domain (combining prescribing and other practice outcomes) is *p* = 0.031. The most common outcome type assessed in these studies was impact on prescribing patterns, which we consider a type of impact on practice. Three of the four studies that reported these outcomes saw improvement from the outreach intervention [34, 36, 38]. The other study did not report an improvement compared to the control (both groups saw an increase in the proportion of ACE inhibitor prescriptions) [33]. This difference may be because the ‘control’ group in this study received evidence-based drug information, which may be more intensive than the control/unexposed groups in the other studies that evaluated this outcome, or because the intervention did not combine several implementation strategies, unlike the other outreach interventions. The *p*-value for the sign test for this outcome sub-domain is 0.125. (Studies with no change or inconsistent results in that outcome domain could not be included in the sign test.)

Both studies that evaluated the impact on other practices saw improvements following the intervention [35, 37] (Tables 4 and 5). The other practices assessed were the use of shared decision-making [37], composition of the team present at the birth of babies < 27 weeks gestational age, use of surfactant within an hour of birth and delivery of trunk into a plastic bag to avoid hypothermia [35]. The *p*-value for the sign test for this outcome sub-domain is 0.25.

##### Impact on policies

Only one study evaluated the impact on policies (around the treatment of premature babies) [35]. The *p*-value for the sign test was 0.5 for this sub-domain. For many of the policies examined, there was little scope for improvement as most units already had the desired policy, and no evidence of difference between the arms was observed. For one policy outcome, units in the control arm were more likely to have the desired policy than those in the intervention arm. However, this difference reflected the pre-intervention differences between the groups, with more intervention arm units introducing the policy during the intervention than control arm units, but not enough to offset the baseline imbalance.

##### Impact on health

Two studies measured the impact on health outcomes, both of which reported small benefits in most health outcomes measured (Tables 4 and 5). The *p*-value for the sign test in this sub-domain was 0.25.

##### Out-takes and outcomes

Only one study reported outputs, out-takes and outcomes [39]. As there was only one study reporting on these domains, the sign test *p*-value was 0.5 for out-takes and outcomes. Among those practitioners who attended an ALLHAT presentation and completed a survey, there were small but consistent increases in expectations and beliefs (out-takes) in line with the results presented compared to before the presentation, and respondents were more likely to report plans to retrain staff, change their own prescription practice and provide lifestyle counselling more often after the intervention than before (we have categorised these as outcomes rather than impacts, as no data is reported on whether these changes actually took place).

### Summary formats for systematic review results

Studies in this category evaluated the use of summary formats for systematic review results, including summary of findings tables, graded-entry formats, GRADE Evidence Profile tables and fishbone diagrams.

#### Included studies

Table 6 provides a summary of the five studies that assessed systematic review summary formats, including study design, setting, length of follow-up, intervention groups assessed, target audience and summary risk of bias assessment. Additional file 7: Table A7.1 provides a description of the interventions studied. All the studies in this category aimed to improve the ability to understand the research results.

**Table 6:** Studies assessing systematic review summary formats (ordered by study design and risk of bias)

Study ID	Design	Setting	Length of follow-up	Intervention groups	Audience	Goal of dissemination	Risk of bias assessment
Opiyo 2013 [40]	Mixed methods: RCT and qualitative interviews	A 1-week national guideline development workshop (‘Child Health Evidence Week’), held in Nairobi, Kenya, between 21 and 25 June 2010	Immediately after the intervention	A: Systematic review alone B: Systematic review plus summary of findings tables C: Graded-entry formats All participants received one tracer-topic with packaging approach A, one with packaging approach B and one with packaging approach C	Healthcare professionals with varied roles in neonatal and paediatric policy and care in Kenya. Sixty-five guideline panel members took part	Ability to understand results and improve usefulness (from systematic reviews on the effectiveness of different neonatal care interventions, including feeding regimens, hand hygiene and Kangaroo care)	Low risk of bias
Vandvik 2012 [41]	RCT comparing two formats of the evidence profile (table A and table B) differing by four features	Online (USA)	Immediately after the intervention	Panellists were randomised to four groups (A1, A2, B1 and B2) based on a 2 by 2 combination of table (A vs B) and clinical question (1 vs 2)	Guideline panellists involved in developing the AT9 guidelines 1A: 18 panellists and 6 editors 1B: 17 panellists and 5 editors 2A: 16 panellists and 5 editors 2B: 16 panellists and 5 editors (28 panellists declined to take part)	Ability to understand results and reduce time spent finding key information (GRADE evidence profiles from a meta-analysis of antithrombotic therapy and prevention of thrombosis using different drugs)	Low risk of bias
Neumann 2018 [42]	This study was a hybrid between a survey and a randomised trial	Grand rounds or clinical meetings in Argentina, Canada, Chile, Costa Rica, Lebanon, Norway, Saudi Arabia, Spain, Switzerland and the USA	Immediately after summaries were given	It compared evidence summaries plus recommendations versus evidence summaries alone. First, participants were randomised to receive either (1) two clinical scenarios involving strong recommendations based on low or very low certainty of evidence or (2) two scenarios involving weak recommendations based on low or very low certainty of evidence. Within each group, respondents were provided with an evidence summary for one scenario and with an evidence summary plus a recommendation for the other scenario. Participants were also randomised in the order in which they received the recommendation, that is, the first or second clinical scenario	Clinicians working primarily in general internal medicine or family medicine. 687 participants were approached, and 496 took part Strong recommendation, recommendation first: 123 Strong recommendation, no recommendation first: 113 Weak recommendation, recommendation first: 131 Weak recommendation, no recommendation first: 128	Ability to understand results and clinicians’ preferences around providing recommendations in addition to evidence summaries (from systematic reviews of the effectiveness of interventions for flu, thrombophilia, nutrition and thrombosis)	Low risk of bias
Rosenbaum 2010a [43]	Two randomised controlled trials: Trial 1 was three arms, and trial 2 was two arms	RCT 1: workshop for newcomers to evidence-based practice RCT2: meeting for members of Continental European Cochrane entities	Immediately after looking at the intervention	Trial 1: • Review with summary of findings table with full formatting • Review with summary of findings table with limited formatting • Review without summary of findings table Trial 2: • Review with summary of findings table • Review without summary of findings table	RCT 1: 72 workshop participants and tutors completed the questionnaires out of approximately 90 people present. These were largely health professionals, many of whom were beginners in evidence-based healthcare and did not have English as their first language. (51 health professionals, 28 researchers) RCT2: The 33 participants were staff members from Cochrane entities, including review group coordinators, trial search coordinators and centre staff. Everybody present participated. Most did not have English as their first language	Ability to understand results and time spent finding key results (from a systematic review of compression stockings for deep vein thrombosis)	Some risk of bias concerns around the possibility of cross-contamination, as participants were in the same room when the different versions of the table were given to participants, so could have seen the alternative version
Gartlehner 2017 [44]	RCT comparing a fishbone diagram to a summary of findings table	Austrian University	Immediately after the intervention	One group was shown a fishbone diagram, and the other group was shown a summary of findings table	77 students who were enrolled in a health sciences bachelor programme or in a health management master’s program	Ability to understand results, utility and time spent finding key information (from a systematic review of pre-operative anaemia management)	Some risk of bias concerns around lack of information on randomisation, blinding and deviations from intended interventions and proportions of missing data across intervention groups

Summary of findings tables present the results from the most important outcomes and quality of evidence scores from systematic reviews, on a single page in a standardised format. GRADE Evidence Profile tables are similar to summary of findings tables, but provide more detailed information about the quality of evidence. Graded entry formats are reports that start with a short summary, followed by a narrative report then the full scientific report, allowing the user to access the main findings quickly, or explore the evidence in more detail if needed. Fishbone diagrams are a way of graphically displaying information, which have been proposed as a way of presenting information about multiple outcomes in a succinct format, that may be simpler than a summary of findings Table [44]. The dissemination activity in all studies was a one-off intervention; however, participants in one study received packs on three topics at the same time [40].

Two of the studies focused on members of clinical guideline development panels [40, 41]. Two studies targeted health professionals [42, 43], with one focusing on general internal or family medicine practitioners [42]. One study also included some health researchers in their sample [43]. One study targeted students in health sciences or health management programmes [44]. The health topics of the systematic reviews that were disseminated were thrombosis [41–43]; neonatal care [40], flu [42], nutrition [42] and pre-operative anaemia [44].

Two studies included participants from low- and middle-income countries (Opiyo 2013: Kenya; and Neumann 2018: Argentina, Costa Rica and Lebanon) [40, 42]. Three studies included participants from high-income countries (Vandvik 2012: USA; Neumann 2018: Canada, Chile, Norway, Saudi Arabia, Spain, Switzerland and the USA; Gartlehner 2017: Austria). The remaining study does not specify which country the study was carried out in [43].

None of the studies relating to summary formats for systematic reviews evaluated impact-type outcomes. One study evaluated the reported recommendation for treatment based on a hypothetical scenario, following receipt of a systematic review summary format [42] (an ‘outcome’). Another assessed whether participants would recommend the format to others and whether they like the format [44]. One study evaluated preferences for specific design features, and between the table formats overall [41]. Several studies assessed out-takes, including knowledge [40, 42, 43], understanding [41, 43, 44], opinions about the summary format such as ease of use, accessibility and value [40, 42–44], time taken to locate information [41, 44] and preferences between different summary formats [40–42].

#### Study quality assessment

The quality of evidence for the studies in this category of intervention was generally good. All studies used a randomised design. Three studies were judged to be at low risk of bias [40–42]. Additional file 8: Table A8.1 shows the GRADE rating of certainty of evidence for all studies in this category.

#### Evidence of effectiveness

##### Out-takes

All five studies reported ‘out-take’ outcomes. Additional file 9: Table A9.1 summarises the results of the studies assessing the effectiveness of summary formats for systematic reviews on outcomes and out-take outcome measures. Table 7 summarises the effect directions for the different outcome types measured in the studies, and Table 8 shows a summary of findings for systematic review summary formats. Taken together, we cannot be confident that the different summary formats studied do improve out-takes from systematic reviews. The sign test *p*-value for out-takes was 0.25. Two studies showed an overall benefit from the summary format [41, 43]. The other studies reported mixed results on ‘out-take’ measures.

**Table 7:**
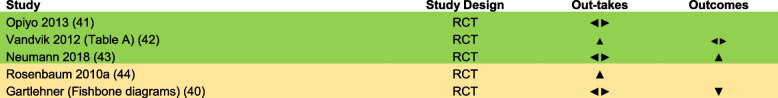
Effect direction plot for systematic review summary formats (ordered by study design and risk of bias) [40–44]

Study design: *RCT* randomised controlled trial, *cRCT* cluster randomised trial

Effect direction: upward arrow ▲ = positive health impact; downward arrow ▼ = negative health impact; sideways arrow ◄► = no change/mixed effects/conflicting findings

Sample size: final sample size (individuals) in the intervention group: large arrow  > 300; medium arrow  50–300; small arrow  < 50

Study quality: denoted by row colour: green = low risk of bias; amber = some concerns; red = high risk of bias

**Table 8:** Summary of findings for systematic review summary formats on outcomes assessed

Outcome assessed	Effect	Number of participants (studies)	Certainty in the evidence^a^
Outcomes	No consistent benefit from systematic review summary formats across the three studies, with one reporting benefit, one detriment and one mixed outcomes	661 participants [41, 42, 44] (3 studies)	Moderate certainty ⊕ ⊕ ⊕ O^b^
Out-takes	No consistent benefit from systematic review summary formats across the studies, although two did find suggestion of overall benefit on out-takes	831 participants [40–44] (5 studies)	Moderate certainty ⊕ ⊕ ⊕ O^b^

^a^Commonly used symbols to describe certainty in evidence in evidence profiles: high certainty (⊕ ⊕ ⊕ ⊕), moderate certainty (⊕ ⊕ ⊕ O), low certainty (⊕ ⊕ OO) and very low certainty (⊕ OOO)

^b^Downgraded by one level for inconsistency

##### Outcomes

Three studies reported ‘outcomes’ [41, 42, 44]. As with out-takes, there is not currently enough evidence to allow us to draw conclusions on whether summary formats for systematic reviews can improve outcomes (Tables 7 and 8), with one study finding mixed results [41], one finding benefit [42] and one finding detriment [44] (the latter compared fishbone diagrams to summary of findings tables). The sign test *p*-value for outcomes was 0.5.

## Discussion

### Summary of key findings

Our systematic review found good evidence that outreach interventions for disseminating clinical research results have a beneficial impact on health outcomes, and moderate certainty evidence of beneficial impact on practice (particularly prescribing), but no evidence of impact on policies. We found no consistent benefit from systematic review summary formats on outcomes or out-takes, despite moderate certainty evidence. There was a dearth of evidence for the knowledge broker and researchers repackaging results intervention categories, although one study at low risk of bias found evidence of the impact of a knowledge broker service on prescribing practice. We found no studies evaluating the effectiveness of different communication approaches (such as tailoring the message to individuals, framing the message in different ways or use of narrative) for sharing the results of clinical studies to professional audiences; however, we did find some qualitative evidence around this which will be reported in a future output from this review.

### How our findings relate to the wider literature

The AHRQ review found multicomponent strategies to be more effective than those that focused on reach, ability or motivation alone [5]. The interventions that we have categorised as ‘outreach’ interventions map most closely to what the AHRQ review describes as multicomponent strategies, as they seek to address a combination of reach, ability and/or motivation, meaning our findings are in line with theirs. Similarly, a systematic review looking at disseminating guidelines found that multi-strategy interventions that include group education and organisational strategies (similar to our ‘outreach’ interventions) were associated with positive significant changes in clinical practice and/or patient outcomes [45]. Another systematic review looking at strategies to increase the uptake of guidelines among musculoskeletal professionals also found multifaceted educational knowledge translation interventions appeared to be effective for improving professional outcomes [46]. The ‘outreach’ interventions in our review include academic detailing (which involves combining several implementation strategies [47]) and similar approaches, which previous research has found to be a valuable way of translating knowledge from comparative research into clinical practice [31]. A systematic review looking at strategies for disseminating research results to US policymakers found that using ‘champions’ or ‘brokers’ was an effective strategy for engaging policymakers [48]. Their concept of ‘champions’ seems similar to the health professionals who delivered outreach interventions, while ‘brokers’ align with knowledge brokers in our included studies. However, their systematic review analysed data thematically, and only one of their studies presented quantitative results relating to health policy. Together, these studies suggest that multicomponent outreach-type interventions are effective at changing practice and improving health outcomes, suggesting that these approaches may be useful where evidence from clinical studies suggests a change in practice is required.

Our results on summary formats for systematic reviews accord with those of a previous systematic review, which found little to no difference in effect in terms of effects on decision-making (impact), out-takes or outcomes for policymakers [49]. A recent mixed methods systematic review evaluated evidence synthesis summary formats for clinical guideline development groups. They found three studies that reported improvements in knowledge or understanding and two with no significant differences between the summary formats tested [50]. Their qualitative results provide useful recommendations on the use of summary formats. Our study had a wider scope in terms of audiences than both these systematic reviews, including health professionals as well as policymakers, but our results are similar.

### Strengths and limitations

The key strength of our study is the extensive search we conducted, screening over 13,000 reports, with inclusive eligibility criteria, including grey literature sources as well as published reports of studies. However, we did not search the CINAHL database, so may have missed some relevant reports, particularly those relating to interventions aimed at nurses and allied health professionals.

Developing the search strategy for this study was challenging, as many publications that are unrelated to our research question use terms relating to our population and interventions of interest, resulting in large numbers of records to screen. The adjacency approach we used was developed to improve the specificity of results of searches, based on numerous test searches. This approach may have excluded a small number of relevant studies that might have been found had we combined terms with AND instead. However, that approach would have resulted in an unmanageable number of records to screen.

The AMEC Integrated Evaluation Framework provided a useful way to conceptualise the different components of evaluations of dissemination activities, allowing us to synthesise the different outcomes measured across the different studies in a meaningful way. While it may not always be realistic to expect dissemination of the results of clinical studies to lead to changes in policy or practice, we included the results of ‘out-take’ and ‘outcome’-type measures alongside ‘impact’ measures reported by studies, allowing us to assess the effectiveness of these interventions across the range of outcomes that have been studied.

Despite our extensive search, the studies we found were limited in both number and often quality, as well as being heterogeneous. This means we were unable to perform meta-analysis or carry out sub-group analyses. It also leaves us unable to draw firm conclusions on the effectiveness of knowledge broker services and researchers repackaging results or provide any results relating to different communication approaches. Our synthesis approach means we are unable to summarise the likely effect size numerically, but can only report consistency of direction of effect.

## Conclusions

Outreach interventions to disseminate clinical research results can lead to changes in clinical practice and improvements in health outcomes. However, outreach interventions are resource intensive [39]. Other effective dissemination approaches are needed, which are feasible for research groups that do not have the resources available to pharmaceutical companies. There was no consistent evidence that systematic review summary formats improve out-takes, such as knowledge or awareness, or outcomes including attitudes to the result of systematic reviews. We found some high-quality evidence that knowledge broker services could lead to changes in prescribing practice, but very low certainty around the effectiveness of researchers repackaging results as dissemination strategies for professional audiences to increase the impact of clinical research. Further, well-designed studies are warranted to evaluate these and other dissemination strategies and thus to guide researchers. Indeed, given clinical trials are so costly and time-consuming, such investment is vital to identify effective and cost-effective ways to disseminate results, so that the potential benefits of trials to patients and the public can be realised.

### Supplementary Information

**Additional file 1. **Search terms, information sources & outcomes of interest. This document shows the search terms, information sources and outcomes of interest for the systematic review.

**Additional file 2. **Results relating to knowledge broker services. This document summarises the results of the review relating to knowledge broker services.

**Additional file 3. **Results relating to researchers repackaging results. This document summarises the results of the review relating to researchers repackaging results for professional audiences.

**Additional file 4: Table A4.1. **Description of the outreach interventions. This table describes the interventions examined in the studies of outreach interventions.

**Additional file 5: Table A5.1. **GRADE rating of certainty of evidence for outreach interventions. This table shows the GRADE ratings of the included studies for outreach interventions.

**Additional file 6: Table A6.1. **Summary of results of studies assessing the effectiveness of outreach interventions. This table summarises the results of included studies that assessed outreach interventions.

**Additional file 7: Table A7.1. **Description of the systematic review summary format interventions. This table describes the interventions examined in the studies of systematic review summary formats.

**Additional file 8: Table A8.1. **GRADE rating of certainty of evidence for systematic review summary formats. This table shows the GRADE ratings of the included studies for systematic review summary formats.

**Additional file 9: Table A9.1. **Summary of results of studies assessing the effectiveness of systematic review summary formats. This table summarises the results of included studies assessing the effectiveness of systematic review summary formats.

## Data Availability

The extracted data used during the current study are available from the corresponding author upon request.

## Abbreviations

ACE: Angiotensin-converting enzyme
AHRQ: Agency for Healthcare Research and Quality
ALLHAT: Antihypertensive and Lipid-Lowering Treatment to Prevent Heart Attack Trial
AMEC: International Association for Measurement and Evaluation of Communication
ASSIA: Applied Social Science Index & Abstracts
AXIS: Appraisal tool for Cross-Sectional Studies
CASP: Critical Appraisal Skills Programme
cRCT: Cluster randomised controlled trial
GRADE: Grading of Recommendations, Assessment, Development and Evaluation
ICTM: Institute of Clinical Trials and Methodology
INVOLVE: NIHR national advisory group on Patient and Public Involvement
MEDLINE: United States National Library of Medicine bibliographic database
MeSH: Medical Subject Headings
MRC: Medical Research Council
PRISMA: Preferred Reporting Items for Systematic Reviews and Meta-Analyses
PROSPERO: International Prospective Register of Systematic Reviews
RCT: Randomised controlled trial
RECAP: REporting Clinical trial results Appropriately to Participants
RoB: Risk of bias
ROBINS-I: Risk Of Bias In Non-randomised Studies—of Interventions
UCL: University College London
