# Supplementary material for: The effectiveness of interventions to disseminate the results of non-commercial randomised clinical trials to healthcare professionals: a systematic review
Source: Implement Sci. 2024 Feb 1;19:8. doi: 10.1186/s13012-023-01332-w (PMC10835915; doi:10.1186/s13012-023-01332-w)
Supplement: Supplementary file 1 — Additional file 1. Search terms, information sources & outcomes of interest. This document shows the search terms, information sources and outcomes of interest for the systematic review. [file 13012_2023_1332_MOESM1_ESM.docx]

# Additional File 1: Search terms and information sources

Contents

[A1.1 Search terms 1](#_Toc143524741)

[A1.1.1 Keyword terms for audiences 1](#_Toc143524742)

[A1.1.2 Keyword terms for communication and dissemination 2](#_Toc143524743)

[A1.1.3 MeSH terms for communication and dissemination 3](#_Toc143524744)

[A1.1.4 Keyword terms for clinical research 3](#_Toc143524745)

[A1.2 Information sources 3](#_Toc143524746)

## A1.1 Search terms

### A1.1.1 Keyword terms for audiences

- Participant*
- Volunteer*
- patient*
- subject*
- consumer*
- caregiver*
- carer*
- relative*
- relation*
- public
- communit*
- lay audience*
- “policy maker*”
- policymak*
- “decision maker”
- commissioner*
- “guideline develop*”
- “health care professional*”
- “health care provider*”
- “health care worker*”
- “health personnel”
- “health professional*”
- “health profession personnel”
- “healthcare personnel”
- “healthcare professional*”
- “healthcare practitioner*”
- “healthcare provider*”
- “healthcare worker*”
- healthworker*
- clinician*
- doctor*
- “medical professional*”
- “medical personnel”
- nurse*
- “health professional*”
- “clinical community”
- professional*
- physician*
- “medical practitioner*”
- practitioner*

### A1.1.2 Keyword terms for communication and dissemination

- “medical information”
- “health communication”
- communicat*
- inform*
- offer*
- disclos*
- return*
- “feeding back”
- feedback
- “feed back”
- provid*
- (deliver* adj4 result*)
- shar*
- notif*
- (disseminat* adj4 result*)
- (presenting adj4 result*)
- (reporting adj4 result*)
- “information dissemination”
- “clinical decision making”
- “family decision making”
- “medical decision making”
- “patient decision making”
- “shared decision making”
- “lay summary”
- “patient education”
- “plain English”
- “persuasive communication”
- “health education”
- “social marketing”
- “public engagement”
- “mass communication”
- “health communication”
- “medical information”
- “information dissemination”
- “dissemination strateg*”
- “information service*”
- “academic detailing”
- guideline*

### A1.1.3 MeSH terms for communication and dissemination

- medical information/
- information dissemination/
- clinical decision making/ or family decision making/ or medical decision making/ or patient decision making/ or shared decision making/
- patient education/
- persuasive communication/
- health education/
- social marketing/
- mass communication/
- information service/

### A1.1.4 Keyword terms for clinical research

- "clinical research"
- “clinical study”
- "cohort study"
- "clinical trial*"
- "meta-analys*"
- "systematic review"
- "epidemiological stud*"
- "randomi* controlled trial"
- "observational study"
- trial
- (clinical adj2 study)

## A1.2 Information sources

We searched the following databases:

- Embase (Ovid interface)
- MEDLINE (Ovid interface)
- PsycINFO (Ovid interface)
- ASSIA
- Cochrane Database of Systematic Reviews

We also searched the following grey literature sources for items:

- ProQuest Dissertations & Theses Global
- The INVOLVE evidence library
- The National Coordinating Centre for Public Engagement’s resources
- Conference proceedings from the Society of Clinical Trials’ annual meetings
- Conference proceedings from the International Clinical Trials Methodology Conference
- Conference proceedings from the Engage conference
- Cochrane Colloquium abstracts
