# Supplementary material for: The effectiveness of interventions to disseminate the results of non-commercial randomised clinical trials to healthcare professionals: a systematic review
Source: Implement Sci. 2024 Feb 1;19:8. doi: 10.1186/s13012-023-01332-w (PMC10835915; doi:10.1186/s13012-023-01332-w)
Supplement: Supplementary file 2 — Additional file 2. Results relating to knowledge broker services. This document summarises the results of the review relating to knowledge broker services. [file 13012_2023_1332_MOESM2_ESM.docx]

Additional File 2: Results relating to knowledge broker services

Contents

[A2.1 Description of included studies 1](#_Toc141950062)

[A2.2 Types of audiences and settings 11](#_Toc141950063)

[A2.3 Outcomes measured 11](#_Toc141950064)

[A2.4 Quality of the evidence 12](#_Toc141950065)

[A2.5 Effectiveness of knowledge broker services 12](#_Toc141950066)

[A2.6 GRADE rating of evidence on knowledge broker interventions 17](#_Toc141950067)

[A2.6 References 21](#_Toc141950068)

# Tables

[Table A2.1 Description of included studies assessing knowledge broker services 3](#_Toc141950191)

[Table A2.2 Overview of the knowledge broker service interventions …………..8](#_Toc141950192)

[Table A2.3: Effectiveness of knowledge broker interventions 15](#_Toc141950193)

[Table A2.4: Effect Direction Plot for Knowledge Broker Services 17](#_Toc141950194)

[Table A2.5: Rating the certainty in evidence on knowledge broker interventions 18](#_Toc141950195)

[Table A2.6: Summary of findings table for knowledge broker interventions 21](#_Toc141950196)

# A2.1 Description of included studies

Table A2.1 provides a summary of the included studies that assessed knowledge broker services, including study design, setting, length of follow-up, intervention groups assessed, target audience and summary risk of bias assessment.

The interventions included in this category are summarised in Table A2.2. They are all ongoing services that aim to identify relevant research, and repackage the results in accessible formats for professional audiences (policymakers and health professionals) who subscribe to their services, aiming to improve ability to understand the research results, and the reach of these results. These repackaged results take a variety of forms in different studies, including evidence-based reviews, position statements, guidelines and policy documents (1), evidence bulletins (2, 3) and email alerts (4). A general feature of the repackaging is to summarise the research and results, making it more concise than a peer-reviewed journal article. As well as sending these summaries to the target audience (by post (3) or email (4)), the knowledge broker services also tended to have websites that contained archives of their summaries (2-4). The geographic scope of the knowledge broker services included varied, from regional (3) to national (1) to international (4). Tanna 2011 and Dormuth 2004 describe regular monthly (4) or bi-monthly (3) editions. The organisations acting as knowledge brokers are not always clearly described, but tend to be academic (3) or non-profit (4) organisations. Based on the information reported, the communication provided does not seem to have been tailored to individual audience members, nor was the approach modified or adapted during the studies. There was no data on how well the approach was implemented in any of the studies.

Table A2.1 Description of studies assessing knowledge broker services (ordered by study design and risk of bias)

| **Study ID** | **Design** | **Setting** | **Length of follow-up** | **Intervention groups** | **Audience** | **Goal of dissemination** | **Risk of Bias assessment** |
| --- | --- | --- | --- | --- | --- | --- | --- |
| Dormuth 2004 (3) | A paired, cluster randomised trial. | Primary care clinics in British Columbia, Canada | 3 months prior to the intervention to two to three months after the intervention | The intervention and control groups were created by grouping an approximate 10% sample of prescribing physicians in 24 local health areas in a paired, cluster randomized design into 12 pairs based on the number of physicians in each area. Local health areas are small geographic regions designated as health analysis regions because of the availability of accurate demographic data. One local health area in each pair was randomly selected and assigned to be in the control group.  For this study the letters were sent to physicians in the intervention group (n = 258) at the same time as to physicians who were not part of the study. Physicians in the control group (n = 241) received the letters 3–8 months later. Letters #7 and #8 on antihypertensive therapy were a special case. They formed a 2-part series and were mailed 10 weeks apart. They were treated as a single intervention; the control group received Letter #7 8 weeks after the intervention group  received Letter #8. | Primary care clinicians in British Columbia, Canada: 258 in the intervention arm, 241 in the control arm. | Reach, ability to understand results | Low risk of bias |
| Tanna 2011 (4) | In 2007–2008, the authors randomized 1,683 subscribers into two different groups receiving select intervention articles, and then they used an online survey to assess both groups on their familiarity with the articles and their acquisition of knowledge. | Worldwide (via email) | Three months | Participants were divided into two groups. Each group then randomly received one of two sets of e-mail alerts (set 1 or set 2) containing an intervention article for each of three months (January,  February, and March 2008). Each month, the intervention article arrived in the monthly NN e-mail alert along with the usual content of 15 to 20 article notifications. Each group received only one intervention article per month; thus, one group’s intervention article became the other’s control article and vice versa. In other words, for each of three consecutive months, half of the subscribers received notification about an intervention article from set 1 while the other half received notification about an intervention article from set 2. | Nephrologists and other physicians. North America: 64%  Europe: 17%  Australia: 6%  Africa: 5%  Asia: 5%  South America: 2% | Reach, ability to understand results | Some risk of bias concerns (low response rate) |
| Johnson 2014 (1) | Survey of stakeholders | Australian states and territories | N/A (cross-sectional) | Knowledge broker service (Heart Foundation’s Active Living program) | Active living professionals in local government, state government, health services, NGOs, health promotion and built environment sectors | Reach, ability to understand results | High risk of bias |
| Dilkes 2008 (2) | Email questionnaire of network members | NI | N/A (cross-sectional) | Health Knowledge Network Evidence Bulletins | Members of the Health Knowledge Network | Reach, ability to understand results | High risk of bias |

Table A2.2 Overview of the knowledge broker service interventions

| **Study** | **Dormuth 2004 (3)** | **Tanna 2011 (4)** | **Johnson 2014 (1)** | **Dilkes 2008 (2)** |
| --- | --- | --- | --- | --- |
| **Intervention name and Implementation strategy/ies (5)** | Therapeutics Letter   - Develop educational materials - Distribute educational materials | Nephrology Now email alerts   - Develop educational materials - Distribute educational materials | Knowledge broker   - Develop educational materials - Distribute educational materials | Health Knowledge Network Evidence Bulletins   - Develop educational materials - Distribute educational materials |
| **Tools/ materials** | Therapeutics Letter is a concise and colourful 2- to 4-page bulletin with an easy-to-read question-and-answer format that is sent to over 6000 physicians in British Columbia.  <https://www.ti.ubc.ca/therapeutics-letter/> | Nephrology Now (NN) is an online, nonprofit service that was created in September 2006 for the worldwide nephrology community. Its current editorial board and creators include practicing nephrologists located across Canada. Subscribers receive a monthly e-mail alert highlighting recently published, clinically relevant articles related to the field of clinical nephrology. The monthly alert includes each article’s title and authors, publication information (the journal in which the article appears and the date of publication), a brief summary of the article, and a link to the article abstract. An independent editorial board selects articles on the basis of their clinical applicability, and the board focuses on articles that have a direct impact on making diagnoses, providing prognoses, and/or guiding treatment. Full free text is available for roughly 35% to 40% of all of NN’s selected articles. | Numerous evidence-based reviews, position statements, guidelines and policy documents to support professionals. | Evidence bulletins were designed to present three layers of information: a cover page outlining the focus and key findings of the review; two to four pages presenting content of the review and contextualising the results; and two to four pages of standardised technical information extracted from the review. |
| **Procedures** | For this study the letters were sent to physicians in the intervention group (n = 258) at the same time as to physicians who were not part of the study.  Physicians in the control group (n = 241) received the letters 3–8  months later. | Each group randomly received one of two sets of e-mail alerts (set 1 or set 2) containing an intervention article for each of three months (January, February, and March 2008). Each month, the intervention article arrived in the monthly NN e-mail alert along with the usual content of 15 to 20 article notifications. Each group received only one intervention article per month; thus, one group’s intervention article became the other’s control article and vice versa. In other words, for each of three consecutive months, half of the subscribers received notification about an intervention article from set 1 while the other half received notification about an intervention article from set 2. | N/I | Network functions comprised: sending Evidence Bulletins to targeted audiences rather than disseminating ad hoc; recruitment strategies for network membership focusing on decision makers to involve diverse audiences including consumer groups, healthcare professionals and policy makers; recruiting key knowledge brokers and encouraging them to forward bulletins to other individuals and organisations; and developing a website for housing bulletins. |
| **Co-interventions** | N/I | Other notifications within Nephrology Now emails. | N/A | N/A |
| **Mode of delivery** | Post | Email | N/I | Website. Unclear how else bulletins were distributed |
| **Who delivered the intervention?** | Therapeutics Initiative of the University of British Columbia | Nephrology Now’s current editorial board and creators include practicing nephrologists located across Canada. | Knowledge broker (Health Foundation) | Key knowledge brokers. Health Knowledge Network. |
| **Where was intervention provided?** | To clinics | Worldwide via email | N/I | N/I |
| **When and how often or much of the intervention was provided?** | Impact of 12 of the first 20 bi-monthly issues evaluated | Monthly | N/I | N/I |
| **Was the intervention tailored?** | No | No | NI | No |
| **Was the approach modified or adapted?** | No | No | N/I | No |
| **How well was the approach delivered?** | N/I | N/I | N/I | N/I |

# A2.2 Types of audiences and settings

Both Tanna 2011 and Dormuth 2004 targeted clinicians as the primary audience for the knowledge broker services, with Dormuth 2004 focusing on primary care clinicians, while Tanna 2011 focused on specialist secondary care clinicians (3, 4). The knowledge broker service described by Johnson 2014 targeted active living professionals in local government, state government, health services, non-governmental organisations, health promotion and the built environment sectors. The target audience for the knowledge broker service described in Dilkes 2008 is unclear. WE have been unable to contact the authors to obtain any further information about this study, beyond what is available in the published abstract.

The knowledge broker service described by Dormuth 2004 covered a wide range of clinical topics, including non-ulcer dyspepsia, peptic ulcer, osteoarthritis and rheumatism, ischemic heart disease, hypertension, sleep, menopause, asthma, anxiety disorders and benign prostatic hyperplasia (3). Other knowledge broker services were more specialised, with Tanna 2011 focusing on nephrology (4) and Johnson 2014 focusing on issues relating to active living (1). It is unclear what topics the knowledge broker service described by Dilkes 2008 covered (2).

The settings for the knowledge broker services were mostly high-income countries. Dormuth 2004 was set in North America (3), and Johnson 2014 was set in Australia (1). Tanna 2011 had an international audience, with most coming from North America and Europe, but also some from Australia, Africa, Asia and South America (4). Dilkes 2008 does not specify where respondents were from (2).

# A2.3 Outcomes measured

Only one of the studies looking at a knowledge broker intervention sought to assess impact-related outcomes: Dormuth 2004 assessed change in prescription patterns (3). Johnson 2014 looked at the proportion of participants who reported that the Heart Foundation’s (knowledge broker) resources that they had used were important to their work (an ‘outcome’)(1). Two studies assessed out-takes: Dilkes 2008 report on participants assessment of the comprehensibility and accessibility of the knowledge broker outputs (2), while Tanna report on participants’ knowledge on the topics included in the knowledge broker service, and familiarity with the trials covered (4).

# A2.4 Quality of the evidence

Two of the studies (Tanna 2011 and Dormuth 2004 (3, 4)) used a randomised controlled trial design, while two were surveys (Johnson 2014 and Dilkes 2008 (1, 2)). The study by Dormuth et al. was judged to be at low risk of bias. There were some risk of bias concerns about the study by Tanna et al because of the low response rate. There were serious risk of bias concerns about the two surveys (Johnson 2014 and Dilkes 2008), however, the information available on these two studies was solely from meeting abstracts, and the concerns mainly relate to lack of sufficient information on issues such as study design, statistical methods, target population, sample frame selection process, measures to address and categorise non-responders, instruments used to measure risk factors and outcome variables, description of data, conflicts of interest and ethical approvals. When contacted, Johnson was unable to provide further information about her study, and we were unable to find contact details for the authors of Dilkes 2008 in order to request further information.

# A2.5 Effectiveness of knowledge broker services

Table A2.3 summarises the results of the studies assessing the effectiveness of outreach interventions on impact, outcomes and out-take outcome measures. Table A2.4 summarises the effect directions for the different outcome types measured in the studies (the effect direction for the out-take from Johnson is excluded from this plot, as it is unclear whether the outcome of ‘wanting further assistance with research evidence’ is beneficial or detrimental). Dilkes 2008 is excluded from these tables as the published abstract contained no results, and we were unable to contact the authors for further information.

The only study at low risk of bias (Dormuth 2004) found evidence that prescription patterns changed more in the direction intended, following receipt of the intervention, compared to in the control group.

Tanna found that the email bulletin Nephrology Now increased familiarity with the articles included in the bulletin, but this did not significantly improve respondents’ knowledge about the articles. As the Effect Direction Plot is focused on point estimates and does not take into account the statistical significance of results, this study is classed as having a benefit for the out-takes domain, but the size of effect for knowledge is very small.

Johnson 2014 found that “the majority” of respondents who had used Heart Foundation resources reported they were important for their work. There is no information about the size of the majority.

With so few studies reporting results, and all using different outcome measures, it is not possible to draw conclusions about whether knowledge broker services are effective or not. With only one study contributing data on each outcome domain, the sign test p-value was 0.5 for each of out-takes, outcomes and impact. The effect directions from all three studies are positive, suggesting there may be benefits from this type of intervention, but more research is needed to explore this further.

Table A2.3: Effectiveness of knowledge broker interventions

| **Study ID** | **Out-takes** | | **Outcomes** | | **Impact** | | **Summary of results** |
| --- | --- | --- | --- | --- | --- | --- | --- |
|  | **Outcome measure** | **Results** | **Outcome measure** | **Results** | **Outcome measure** | **Results** |  |
| Dormuth 2004 (3) | N/A | N/A | N/A | N/A | Number of newly treated patients who received that letter’s analysis drug before versus after the intervention | The preference for the analysis drugs was 1.3 times more in the predicted direction in the intervention group of physicians  than in the control group (95% confidence interval 1.13–1.52) | Prescriptions of the analysis drugs significantly increased in the intervention arm compared to the control arm. |
| Tanna 2011 (4) | Familiarity (*On a scale of 1 to 5, where 1 is totally unfamiliar and 5 is very familiar, how would you rank your familiarity with the following article?)* | +0.23 ±0.087 (95% CI 0.06 -0.41) | N/A | N/A | N/A | N/A | The Nephrology Now email alerts significantly improved familiarity with the relevant articles, but this did not lead to an improvement in knowledge acquisition. |
|  | Knowledge acquisition (*to what degree they agreed (1 = strongly agree, 5 = strongly disagree) with a statement of fact from the intervention article)* | +0.03±0.083 (95% CI -0.13 to 0.2) |  |  |  |  |  |
| Johnson 2014 (1) | Proportion of participants who reported wanting further assistance with research evidence | “Almost 60%” | Of those who had used Heart Foundation resources, did they report they were important to their work | “Majority” | N/A | N/A | The majority of those who reported having used the Heart Foundation resources reported the resources were important to their work. Most participants also reported wanting further assistance with research evidence. |

Table A2.4: Effect Direction Plot for Knowledge Broker Services

| **Study** | **Study Design** | **Out-takes** | **Outcomes** | **Impact on Prescribing** | **RoB** |
| --- | --- | --- | --- | --- | --- |
| Dormuth 2004 | cRCT |  |  | ▲ | aLow |
| Tanna 2011 | RCT | ▲ |  |  | bSomeConcerns |
| Johnson 2014 | Observational |  | ▲ |  | cHigh |
|  |  |  |  |  |  |
| LEGEND |  |  |  |  |  |
| Study design: RCT: Randomised Controlled Trial; CRCT: Cluster Randomised Trial; etc | | | | |  |
| Effect direction: upward arrow ▲= positive health impact, downward arrow ▼= negative health impact, sideways arrow ◄►= no change/mixed effects/conflicting findings | | | | | |
| Sample size: Final sample size (individuals) in intervention group Large arrow ▲ >300; medium arrow ▲ 50-300; small arrow ▲ <50 | | | | | |
| Study quality: denoted by row colour: green = low risk of bias; amber = some concerns; red = high risk of bias | | | | | |

# A2.6 GRADE rating of evidence on knowledge broker interventions

Table A2.5: Rating the certainty in evidence on knowledge broker interventions

| **GRADE domain** | **Judgement** | **Concerns about certainty domains** |
| --- | --- | --- |
| **Impact on practice - prescribing** | | |
| Risk of bias | The only study contributing to this outcome was at low risk of bias. | Not suspected |
| Indirectness | The population, intervention, comparator and outcomes in the included study are directly relevant for the question of this review. | Not suspected |
| Imprecision | In this cluster randomised trial, data were collected about prescriptions for 748 newly-treated patients of intervention physicians, and 620 patients of control physicians. The confidence interval excludes harm or no effect. | Not suspected |
| Inconsistency | Not applicable, as only one outcome available for this impact domain | Not suspected |
| Publication bias | We carried out a comprehensive search for studies, but only found one study reporting the impact on practice of knowledge broker interventions. | Not suspected |
| Large effects | Not applicable | Not upgraded |
| Dose response | Not applicable | Not upgraded |
| Opposing plausible residual bias and confounding | Not applicable | Not upgraded |
| **Impact on outcomes** | | |
| Risk of bias | The only study reporting outcomes was judged to be at high risk of bias because insufficient information about the methods or results were reported in the abstract, and further information was not available from the author. | Serious concerns - downgraded |
| Indirectness | The population, intervention and outcome in the included study are directly relevant for the question of this review. | Not suspected |
| Imprecision | The study reports on data from 398 respondents. No confidence interval or similar estimate of precision is reported. | Suspected |
| Inconsistency | Not applicable, as only one outcome available for this impact domain | Not suspected |
| Publication bias | We carried out a comprehensive search for studies, but only found one study reporting the impact on outcomes of knowledge broker interventions. | Not suspected |
| Large effects | Not applicable | Not upgraded |
| Dose response | Not applicable | Not upgraded |
| Opposing plausible residual bias and confounding | Not applicable | Not upgraded |
| **Impact on out-takes** | | |
| Risk of bias | The only study reporting information on out-takes was judged to be at some risk of bias because of a low response rate making it unclear how generalisable the results were to the target population. | Downgraded one level. |
| Indirectness | The population, intervention, comparator and outcome in the included study are directly relevant for the question of this review. | Not suspected |
| Imprecision | The study reports data from 803 respondents. The confidence interval for familiarity excludes harm or no effect, but the confidence interval of knowledge includes both harm and benefit, but is not very large. As this study is of a reasonable size, it has not been downgraded for imprecision. | Not downgraded. |
| Inconsistency | The effect direction is the same for both familiarity and knowledge, however the effect size ranges from 0.03 to 0.23 | Downgraded one level |
| Publication bias | We carried out a comprehensive search for studies, but only found one study reporting the impact on out-takes of knowledge broker interventions. | Not suspected |
| Large effects | Not applicable | Not upgraded |
| Dose response | Not applicable | Not upgraded |
| Opposing plausible residual bias and confounding | Not applicable | Not upgraded |

Table A2.6: Summary of findings table for knowledge broker interventions

| **Outcome** | **Effect** | **Number of participants (studies)** | **Certainty in the evidence*** |
| --- | --- | --- | --- |
| Impact on practice (prescribing) | The knowledge broker service led to changes in prescription in the direction predicted (risk ratio 1.3) | One RCT with data from 748 newly-treated patients of intervention physicians, and 620 patients of control physicians post-intervention  (1 study) | High certainty ⊕⊕⊕⊕ |
| Outcomes | The majority of respondents reported the resources were important for their work | One observational study with data from 398 respondents.  (1 study) | Very low certainty^†^  ⊕OOO |
| Out-takes | The effect direction suggests improvements in familiarity with the research, with a much smaller (non-statistically significant) effect on knowledge | One observational study with data from 803 respondents.  (1 study) | Low certainty^‡^  ⊕⊕OO |

*Commonly used symbols to describe certainty in evidence in evidence profiles: high certainty ⊕⊕⊕⊕, moderate certainty ⊕⊕⊕O, low certainty ⊕⊕OO and very low certainty ⊕OOO.

^†^ Downgraded by one level for risk of bias concerns

^‡^Downgraded by one level for risk of bias concerns around response rate, and one level for inconsistency

# A2.6 References

1. Johnson B. Making the link between policy makers, practitioners and research: Awareness, use and perceptions of active living professionals. Journal of Science and Medicine in Sport. 2014;1):e8.

2. Dilkes H, Hill S, Ryan R. Evidence for improving communication and participation: evaluation of Evidence Bulletin knowledge transfer. Cochrane Colloquiem; Freiburg2008.

3. Dormuth CR, Maclure M, Bassett K, Jauca C, Whiteside C, Wright JM. Effect of periodic letters on evidence-based drug therapy on prescribing behaviour: a randomized trial. CMAJ. 2004;171(9):1057-61.

4. Tanna GV, Sood MM, Schiff J, Schwartz D, Naimark DM. Do e-mail alerts of new research increase knowledge translation? A "Nephrology Now" randomized control trial. Acad Med. 2011;86(1):132-8.

5. Powell BJ, Waltz TJ, Chinman MJ, Damschroder LJ, Smith JL, Matthieu MM, et al. A refined compilation of implementation strategies: results from the Expert Recommendations for Implementing Change (ERIC) project. Implementation Science. 2015;10(1):21.
