# Supplementary material for: The effectiveness of interventions to disseminate the results of non-commercial randomised clinical trials to healthcare professionals: a systematic review
Source: Implement Sci. 2024 Feb 1;19:8. doi: 10.1186/s13012-023-01332-w (PMC10835915; doi:10.1186/s13012-023-01332-w)
Supplement: Supplementary file 3 — Additional file 3. Results relating to researchers repackaging results. This document summarises the results of the review relating to researchers repackaging results for professional audiences. [file 13012_2023_1332_MOESM3_ESM.docx]

Additional file 3: Results relating to researchers repackaging study results

Contents

[A3.1 Description of included studies 1](#_Toc141950798)

[A3.2 Types of audiences and settings 9](#_Toc141950799)

[A3.3 Outcomes measured 9](#_Toc141950800)

[A3.4 Quality of the evidence 9](#_Toc141950801)

[A3.5 Effectiveness of researchers repackaging results 10](#_Toc141950802)

[A3.6 GRADE rating of evidence on researchers repackaging results 20](#_Toc141950803)

[A3.6 References 23](#_Toc141950804)

Tables

[Table A3.1 Description of studies assessing researchers repackaging study results ………………………………………………………………………………..3](#_Toc141950825)

[Table A3.2a Overview of researchers repackaging research results interventions in included studies 6](#_Toc141950826)

[Table A3.2b Overview of researchers repackaging research results interventions in included studies (ctd.) 8](#_Toc141950827)

[Table A3.3: Effectiveness of repackaging interventions 14](#_Toc141950828)

[Table A3.4: Effect Direction Plot for Repackaging interventions 20](#_Toc141950829)

[Table A3.5: Rating the certainty in evidence on repackaging interventions 21](#_Toc141950830)

[Table A3.6: Summary of findings table for researchers repackaging results 23](#_Toc141950831)

# A3.1 Description of included studies

Table A3.1 provides a summary of the included studies that assessed researchers repackaging study results, including study design, setting, length of follow-up, intervention groups assessed, target audience and summary risk of bias assessment. Four studies involved researchers repackaging study results in some way. These interventions are described in Table A3.2. The results were repackaged in different forms in different studies, including an online continuing medical education video of a roundtable discussion (1), a face-to-face workshop (2), an abstract, a plain language summary, an audio podcast, and the transcript of the audio podcast (3), different versions of policy briefs (4), and a one-page summary (5). The aim of the repackaging was to improve ability to understand the research results. The interventions were all delivered as one-off engagements with the target audiences, although Masset 2013 drew participants from the contact lists of a knowledge broker organisation. None of the interventions were tailored for individual audience members, the approaches were not modified or adapted during the studies, and no information was reported on how well the approach was delivered. Maguire 2015 is excluded from the tables on effectiveness and GRADE rating, as no results were available for this study (3).

Table A3.1 Description of studies assessing researchers repackaging study results (ordered by study design and risk of bias)

| **Study ID** | **Design** | **Setting** | **Length of follow-up** | **Intervention groups** | **Audience** | **Goal of dissemination** | **Risk of Bias assessment** |
| --- | --- | --- | --- | --- | --- | --- | --- |
| Maguire 2015 (3) | Random assignment to one of four forms of summary of a systematic review. Then answered questions about the key findings. | N/I | Immediately after reading/listening to the summary | 4 types of summary:   1. Abstract 2. Plain language summary 3. Audio podcast   Transcript of the podcast | People who were on University and organisational mailing lists | Ability to understand results | Some risk of bias concerns |
| Masset 2013 / Beynon 2012 (4, 6) | RCT | International study carried out online | 3 months | (T1) standard policy brief  (T2) policy brief with director’s commentary  (T3) policy brief with research fellow’s commentary and (Control) a ‘placebo’ policy brief (on a different topic) | People on contact lists held by  the Knowledge Services Department at the Institute of Development Studies (IDS) and  International Initiative for Impact Evaluation (3ie’s) | Ability to understand results | Some risk of bias concerns |
| Williams 2010 (5) | Pre/post intervention surveys | Surveys posted to paediatricians in Australasia | Post-survey was sent along with the evidence summary | All participants were sent a one-page evidence summary | 215 paediatricians in Australasia | Ability to understand results | Some risk of bias concerns |
| Larkin 2017 (1) | Pre/post- intervention questionnaire | Online | Immediately before and after participants were exposed to the intervention | Online continuing Medical Education activity | Primary care physicians and diabetologists/ endocrinologists | Ability to understand results | Serious risk of bias concerns |
| Baker 2015 (2) | Pre/post-intervention poll of attendees. | Face-to-face workshops for public health practitioners in Canada. | Immediately before and after the presentation. | Attendees at workshops communicating the results of a systematic review | Public health practitioners from three Canadian public health units | Ability to understand results | Serious risk of bias concerns |

Table A3.2a Overview of researchers repackaging research results interventions in included studies

| **Study** | **Maguire 2015 (3)** | | | | **Williams 2010 (5)** | **Larkin 2017 (1)** | **Baker 2015 (2)** |
| --- | --- | --- | --- | --- | --- | --- | --- |
| **Intervention name and Implementation strategy/ies (7)** | **Abstract** | **Plain language summary**  Develop educational materials | **Online continuing medical education**  Develop educational materials | **Transcript of podcast**  Develop educational materials | **Evidence summary**  Develop educational materials | **Online continuing medical education**  Develop educational materials | **Workshop**  Develop educational materials |
| **Tools/ materials** | Abstract of the systematic review | Plain language summary of the systematic review | The CME activity was developed as an online video roundtable discussion between 3 experts in Type 2 Diabetes management. | Transcript of the audio podcast | A one page summary of the results of a systematic review | The CME activity was developed as an online video roundtable discussion between 3 experts in Type 2 Diabetes management. | Workshop presentation |
| **Procedures** | After answering a question about what they thought the key finding of the review would be they were randomly assigned to one of the summaries. They were then asked to spend no more than 15 minutes reading or listening to the summary, before answering again the question about the key review findings and to indicate whether they would want to read the full Cochrane Review | | | | The Evidence Summary was sent with the post-intervention survey | N/I | N/I |
| **Co-interventions** | N/A | N/A | N/I | N/A | N/A | N/I | N/I |
| **Mode of delivery** | N/I | N/I | Online | N/I | Post | Online | Face-to-face workshop |
| **Who delivered the intervention?** | N/I | N/I | Experts in type 2 diabetes management. | N/I | N/I | Experts in type 2 diabetes management. | N/I |
| **Where was intervention provided?** | N/I | N/I | Online | N/I | N/I | Online | Public health units where participants worked |
| **When and how often or much of the intervention was provided?** | One summary (up to 15 minutes of reading) | One summary (up to 15 minutes of reading) | The activity launched on November 19, 2015 and data were collected through January 29, 2016.  1 session. | One summary (up to 15 minutes of reading) | One-off | The activity launched on November 19, 2015 and data were collected through January 29, 2016.  1 session. | One-off |
| **Was the intervention tailored?** | No | No | No | No | No | No | N/I |
| **Was the approach modified or adapted?** | No | No | No | No | No | No | N/I |
| **How well was the approach delivered?** | N/I | N/I | N/I | N/I | N/I | N/I | N/I |

Table A3.2b Overview of researchers repackaging research results interventions in included studies (ctd.)

| **Study** | **Masset 2013/Beynon 2012 (4, 6)** | | | |
| --- | --- | --- | --- | --- |
| **Intervention name** | T1: standard policy brief | T2: policy brief with director’s commentary | T3: Policy brief with research fellow’s commentary | Placebo policy brief |
| **Tools/ materials** | The policy brief was a colour leaflet containing an introduction to the problem of world hunger, a description of the methodology employed by the review and a set of conclusions and policy implications. | | | |
|  | The first version was a basic policy  brief of three pages. | The second version was identical to the first version with the addition  of a concluding opinion piece written by a sector expert and director of the institution  conducting the review (five pages total). | The third version was identical to the second  version, but the final commentary was credited to an unnamed research fellow rather than  to the director of the institute. | A ‘placebo’ brief that did not communicate any knowledge to its readers relevant  to forming opinions about the policies in question. |
| **Procedures** | Baseline participants were contacted by email and provided with a link to the relevant communication intervention for their group. | | | |
| **Co-interventions** | N/A | N/A | N/A | N/A |
| **Mode of delivery** | Provided with a link by email | | | |
| **Who delivered the intervention?** | Knowledge broker | | | |
| **Where was intervention provided?** | Online | | | |
| **When and how often or much of the intervention was provided?** | One-off (but invited people were mostly subscribes to knowledge broker service) | | | |
| **Was the intervention tailored?** | No | | | |
| **Was the approach modified or adapted?** | No | | | |
| **How well was the approach delivered?** | N/I | | | |

# A3.2 Types of audiences and settings

The target audience of Williams 2010, Larkin 2017 and Baker 2015 were all health professionals. Williams 2010 targeted paediatricians (5), while Larkin 2017 targeted primary care physicians, diabetologists and endocrinologists (1), Baker targeted public health professionals (2) and Masset 2013 targeted development professionals (mostly based at international aid organisations and non-governmental organisations)(4). The target audience of Maguire 2015 is unclear, specifying only people on university and organisational mailing lists (3). The health topics of the repackaged studies included urinary tract infections (Williams 2010), diabetes (Larkin 2017), physical activity (Baker 2015), child nutrition (Masset 2013) and depression, weight loss, gastrointestinal problems and reducing sitting (Maguire 2015) (1-5). Williams 2010 and Baker 2015 were both set in high-income settings (Australasia and Canada respectively) (2, 5), Masset 2013 was an international study across high and low and middle-income settings, with data collection carried out online (4), while the setting of Maguire 2015 and Larkin 2017 is unclear, with data collection taking place online and little information given about the geographical location of participants (1, 3).

# A3.3 Outcomes measured

None of the studies relating to repackaging of research results looked at impact-related outcomes. Two studies reported ‘Outcome’-type outcomes: Maguire et al. report on desire to read the full review (3), while Williams et al. look at reported intention to change practice (5). All the studies that looked at the repackaging of research results by researchers measured out-takes, including knowledge (Larkin 2017, Baker 2015 and Williams 2010) (1, 2, 5), familiarity (Larkin 2017) (1), beliefs (Baker 2015) (2), understanding (Maguire 2015) (3) and views on the strength of evidence (Masset 2013) (4).

# A3.4 Quality of the evidence

Overall, the quality of evidence on researchers repackaging research results was lower than for the previous two categories. Two of the studies looking at researchers repackaging research results randomised participants to one of four forms of summary (Maguire 2015 and Masset 2013 (3, 4)). The remaining studies used pre- and post-intervention questionnaires (Larkin 2017, Baker 2015 and Williams 2010 (1, 2, 5)). None of the studies on repackaging research results were considered to be at low risk of bias. There were some risk of bias concerns around Maguire 2015 due to lack of information on potential bias due to deviations from randomisation, lack of information on missing data, and the potential for measurement bias. This was a conference abstract, and the author of this study was contacted for further information, but no further information was received. There were some risk of bias concerns around Masset 2013 due to missing data. There were also some risk of bias concerns about Williams 2010 due to lack of sample size justification, lack of information on the instruments used to measure risk factors and outcome variables, and lack of information on non-responders. Larkin 2017 was judged to be at serious risk of bias due to the potential for selection bias, as inclusion in the analysis was based on having answered all the test questions, which could be related to how well the respondent understood the information conveyed in the intervention, with those who found the intervention less comprehensible potentially being more likely to not answer all the questions, favouring the experimental arm. No data was provided in the abstract or poster around how many participants were excluded for this reason. Baker 2015 was also judged to be at serious risk of bias as the meeting abstract contained no sample size justification, it was unclear how representative of the target population those who attended the workshop were, no measures were undertaken to address and categorise non-responders, no information was provided on the whether the instruments used to measure risk factors and outcome variables had been previously trialled or tested, lack of description of data, no information about response rates, no information about conflicts of interest or ethical approvals. We were unable to find contact information for the authors.

# A3.5 Effectiveness of researchers repackaging results

Table A3.3 summarises the results of the studies assessing the effectiveness of repackaging interventions on outcomes and out-take outcome measures. Table A3.4 summarises the effect directions for the different outcome types measured in the studies. Maguire 2015 is excluded from these tables as the published abstract contained no results, and we were unable to contact the authors for further information.

Only one study, Williams 2010, looked at the effect of repackaging interventions on outcomes (reported likelihood of ordering the types of scans that the intervention focused on, in different scenarios). This study found that, where reported pre-test practice was not 100% in line with the recommendations of the intervention, post-test reported intended practice changed to be more in line with the recommendations. The differences in proportion reporting they would use the recommended approach was between 1% to 13%, depending on the outcome, mostly around 10% (5).

All four studies that reported results on repackaging interventions reported on out-takes. Masset 2013 was the only RCT conducted on these interventions. This found inconsistent results, with the briefing papers increasing the proportion of participants who had an opinion on the strength of evidence (by 20-25%), and, to a lesser extent, the proportion having an opinion on the effectiveness of the agricultural interventions the policy briefs focused on. However, it found no difference in participants’ ratings of either the strength of evidence, or the effectiveness of the interventions, compared to those who received the control policy brief (4). The other studies which looked at out-takes all reported benefits from the repackaging interventions on out-takes. Williams 2010 found that the proportion of respondents who answered questions correctly increased by between 27% to 47% following the intervention (5). Larkin found a significant improvement in mean test score post intervention, with the increase in percent of people getting individual questions right ranging from 5% to 36%, depending on the question and the specialty of respondents (1). Baker 2015 looked at the effect of repackaging interventions on beliefs, with changes ranging from 1.5% to 68% (2).

While the available evidence mostly points towards repackaging interventions potentially having an effect on out-takes, the relatively few studies, and the quality limitations of these studies, makes it hard to be confident in this approach, and more evidence is needed. The sign p-value for the out-takes domain was 0.125. With only one study contributing evidence on the outcomes domain, with some risk of bias concerns, there is even less evidence in this area. The sign p-value for outcomes was 0.5.

Table A3.3: Effectiveness of repackaging interventions

| **Study ID** | **Out-takes** | | **Outcomes** | | **Summary of results** |
| --- | --- | --- | --- | --- | --- |
|  | **Outcome measure** | **Results** | **Outcome measure** | **Results** |  |
| **Williams 2010 (5)** | Proportion who correctly estimated likelihood of vesicoureteric reflux | Increased from 58% pre to 85% post | Median reported likelihood of ordering an ultrasound | Was 100% both pre and post | The Evidence Summary was associated with improvements in knowledge about test performance, and also reported intended changes in practice (where current practice was not already at 100%). |
|  | Proportion who correctly estimated likelihood of renal damage | Increased from 6% pre to 39% post | Median reported likelihood of ordering a VCUG for 2-month-old child | Was 100% both pre and post |  |
|  | Proportion who correctly estimated the sensitivity of ultrasound to detect renal damage | Increased from 12% pre to 49% post | Median reported likelihood of ordering a VCUG for 3-year-old child | Decreased from 55% pre to 45% post |  |
|  | Proportion who correctly estimated the test performance of ultrasound for detection of reflux | Increased from 10% pre to 56% post | Median reported likelihood of ordering a VCUG for 6-year-old child | Decreased from 3% pre to 2% post |  |
|  | Proportion who correctly estimated the test performance of DMSA detection of reflux | Increased from 6% pre to 53% post | Median reported likelihood of ordering a DMSA scan for children with febrile UTIs | Increased for children aged 2 months from 80% pre to 90% post.  Increase for children aged 3 years from 60% pre to 71% post  Increased for children aged 6 years from 17% pre to 30% post |  |
|  | Proportion who correctly estimated the test performance of VCUG detection of renal damage | Increased from 3% pre to 36% post | Median reported likelihood of ordering a DMSA scan for children with non-febrile UTIs | Increased for children aged 2 months from 38% pre to 44% post  Increase for children aged 3 years from 10% pre to 15% post  Increased for children aged 6 years from 4% pre to 5% post |  |
| **Masset 2013 (4)** | The fraction of respondents having an opinion regarding the strength of evidence of four agricultural interventions on nutrition (at 3 months) | The policy brief increased the percentage of respondents with an opinion on strength of evidence by 20–25 decimal points. | N/A | N/A | Policy briefs were effective at increasing the proportion of respondents having an opinion about the strength of evidence of the agricultural interventions, and to a lesser extent having an opinion on the effectiveness of these interventions, but did not have an effect on the average rating of the strength of evidence nor average rating of effectiveness of the interventions at 3 months. |
|  | The fraction of respondents having an opinion regarding the effectiveness of four agricultural interventions on nutrition (at 3 months) | The policy brief only modestly increased the number of respondents with an opinion regarding the effectiveness of the interventions with the exception of biofortification (which was 18-27) |  |  |  |
|  | Respondents’ ratings of strength of evidence of each intervention (at 3 months) | With the exception of a half point reduction in the strength of effectiveness rating of biofortification, the policy brief produced no changes in average respondents’ scores. We could not find a differential impact of the brief with or without an additional commentary by the director of the institute or by a research fellow for any of the four interventions considered. |  |  |  |
|  | Respondents’ ratings of effectiveness of each intervention (at 3 months) | The policy brief produced no changes in average respondents’ scores. We could not find a differential impact of the brief with or without an additional commentary by the director of the institute or by  a research fellow for any of the four interventions considered. |  |  |  |
| **Larkin 2017 (1)** | Improvement in mean score | Cramer’s V=0.215 for primary care physicians, and V=0.131 for diabetologists / endocrinologists (p<0.05 for both) | N/A | N/A | Participants’ mean score for correctly answered questions significantly increased following the intervention. |
|  | Recognised the effect of an SGLT2 inhibitor on body weight | 36% increase in primary care physicians  29% increase in diabetologists / endocrinologists |  |  |  |
|  | Recognised recent clinical trial data on cardiovascular outcomes for an SGLT2 inhibitor | 57% post vs 42% pre intervention for primary care physicians  74% post vs 69% pre intervention for diabetologists / endocrinologists |  |  |  |
|  | Correctly translated recent clinical trial data into appropriate patient education point related to genital mycotic infections | 32% more post intervention for primary care physicians  24% more post intervention for diabetologists / endocrinologists |  |  |  |
| **Baker 2015 (2)** | Strongly agreed ‘Community-wide interventions effectively increase population levels of physical activity’ | Reduced from 3% before the presentation to 1.5% after the presentation | N/A | N/A | The workshop led to significant changes in beliefs of attendees about the effectiveness of the intervention reviewed (mostly in the direction of the results of the systematic review). |
|  | Agreed ‘Community-wide interventions effectively increase population levels of physical activity’ | Reduced from 36% before the presentation to 3% after the presentation |  |  |  |
|  | Increased their belief in the intervention by one level | 6% |  |  |  |
|  | Unchanged in their view about the intervention | 26% |  |  |  |
|  | Reduced their belief in the intervention by one or more level | 68% |  |  |  |

Table A3.4: Effect Direction Plot for Repackaging interventions

| **Study** | **Study Design** | **Out-takes** | **Outcomes** |
| --- | --- | --- | --- |
| Masset 2013 (4) | RCT | ◄► |  |
| Williams 2010 (5) | Pre/post intervention surveys | ▲ | ▲ |
| Larkin 2017 (1) | Pre/post intervention surveys | ▲ |  |
| Baker 2015 (2) | Pre/post intervention surveys | ▲ |  |
| LEGEND |  |  |  |
| Study design: RCT: Randomised Controlled Trial; CRCT: Cluster Randomised Trial; etc | | | |
| Effect direction: upward arrow ▲= positive health impact, downward arrow ▼= negative health impact, sideways arrow ◄►= no change/mixed effects/conflicting findings | | | |
| Sample size: Final sample size (individuals) in intervention group Large arrow ▲ >300; medium arrow ▲ 50-300; small arrow ▲ <50 | | | |
| Study quality: denoted by row colour: green = low risk of bias; amber = some concerns; red = high risk of bias | | | |

# A3.6 GRADE rating of evidence on researchers repackaging results

Table A3.5: Rating the certainty in evidence on repackaging interventions

| **GRADE domain** | **Judgement** | **Concerns about certainty domains** |
| --- | --- | --- |
| **Impact on outcomes** | | |
| Risk of bias | The only study contributing to this domain was a non-randomised study with some risk of bias concerns due to lack of sample size justification, lack of information on the instruments used to measure risk factors and outcome variables, and lack of information on non-responders. | Downgraded one level |
| Indirectness | The population, intervention and outcome are directly relevant to the question of this review. | Not suspected |
| Imprecision | The study has a moderate sample size (215 respondents), and there is substantial variability in reported ordering practice across several of the outcomes (for example, the interquartile range for ordering a VCUG scan for a three year old ranged from 4-97%). | Downgraded one level |
| Inconsistency | The direction and effect size on this outcome was consistent across the measured outcomes, ranging from 1-13%, with most outcomes around 10%. | Not suspected |
| Publication bias | We carried out a comprehensive search for studies, but only found one study reporting the impact on practice of knowledge broker interventions. | Not suspected |
| Large effects | Not applicable – only a small effect size was observed | Not upgraded |
| Dose response | Not applicable | Not upgraded |
| Opposing plausible residual bias and confounding | Not applicable | Not upgraded |
| **Impact on out-takes** | | |
| Risk of bias | Of the four studies that contribute data on this outcome, only one uses a randomised design, and all are affected by some or high levels of risk of bias concern. | Serious concern: downgraded by one level |
| Indirectness | The populations, interventions and outcomes are directly relevant to the question of this review. | Not suspected |
| Imprecision | The included studies have a moderate sample size (data from at least 580 participants), and the results from each study seem reasonably precise, judging from the information available. | Not suspected |
| Inconsistency | The only RCT contributing data to this domain found inconsistent results, while the other studies report results that are reasonably consistent | Downgraded by one level |
| Publication bias | We carried out a comprehensive search for studies, and found four studies reporting results on out-takes. | Not suspected |
| Large effects | Not applicable | Not upgraded |
| Dose response | Not applicable | Not upgraded |
| Opposing plausible residual bias and confounding | Not applicable | Not upgraded |

Table A3.6: Summary of findings table for researchers repackaging results

| **Outcome** | **Effect** | **Number of participants (studies)** | **Certainty in the evidence*** |
| --- | --- | --- | --- |
| Outcomes | There is a suggestion of a small benefit of repackaging interventions on reported intended behaviour, in the range of 1-13%. | 215 respondents  (1 study) | Very low certainty^†^  ⊕OOO |
| Out-takes | There is a suggestion of a moderate benefit of repackaging interventions on out-takes | At least 580 respondents (number of respondents not reported in Larkin)  (4 studies) | Very low certainty  ⊕OOO^‡^ |

*Commonly used symbols to describe certainty in evidence in evidence profiles: high certainty ⊕⊕⊕⊕, moderate certainty ⊕⊕⊕O, low certainty ⊕⊕OO and very low certainty ⊕OOO.

^†^ Downgraded by one level for risk of bias concerns and one level for imprecision

^‡^Downgraded by one level for risk of bias concerns, and one level for inconsistency

# A3.6 References

1. Larkin A, LaCouture M, Le A. What does recent data related to SGLT2 inhibitors mean for my patients? Effect of online medical education on physician knowledge and competence. Endocrine Reviews Conference: 99th Annual Meeting of the Endocrine Society, ENDO. 2017;38(3 Supplement 1).

2. Baker P, Francis D, Demant D. Does presenting the findings of a review change public health decision-makers’ view on an intervention? Cochrane Colloquiem; Vienna2015.

3. Maguire L, Clarke M, Tully M. Reading or listening to review summaries - which method will produce greater understanding of the key outcomes in a cochrane review? 3rd International Clinical Trials Methodology Conference; Glasgow: Trials; 2015.

4. Masset E, Gaarder M, Beynon P, C C. What is the impact of a policy brief? Results of an experiment in research dissemination. Journal of Development Effectiveness. 2013;5:50-63.

5. Williams GJ, Sureshkumar P, Wheeler D, Craig JC. Paediatrician's responses to an evidence summary about renal tract imaging tests in children after urinary tract infection. Arch Dis Child. 2010;95(4):271-5.

6. Beynon P, Chapoy C, Gaarder M, E M. What difference does a policy brief make? Full report of an IDS, 3ie, Norad study. Institute of Development Studies and the International Initiative for Impact Evaluation (3ie); 2012.

7. Powell BJ, Waltz TJ, Chinman MJ, Damschroder LJ, Smith JL, Matthieu MM, et al. A refined compilation of implementation strategies: results from the Expert Recommendations for Implementing Change (ERIC) project. Implementation Science. 2015;10(1):21.
