# Supplementary material for: The effectiveness of interventions to disseminate the results of non-commercial randomised clinical trials to healthcare professionals: a systematic review
Source: Implement Sci. 2024 Feb 1;19:8. doi: 10.1186/s13012-023-01332-w (PMC10835915; doi:10.1186/s13012-023-01332-w)
Supplement: Supplementary file 4 — Additional file 4: Table A4.1. Description of the outreach interventions. This table describes the interventions examined in the studies of outreach interventions. [file 13012_2023_1332_MOESM4_ESM.docx]

# Additional File 4: Description of the outreach interventions

**Table A4.1: Description of the outreach interventions** (ordered by study design and risk of bias)

| **Study** | **Skoglund 2013 (1)** | **Ludden 2018 (2)** | **Acolet 2011 (3)** | **Bernal-Delgado 2002 (4)** | **Stafford 2010 & Bartholomew 2009 (4, 5)** | **Majumdar 2003(6)** |
| --- | --- | --- | --- | --- | --- | --- |
| **Intervention name and Implementation strategy/ies (7)** | **Motivational Interviewing (MI)**   - Conduct educational meetings | **FLOW**   - Conduct educational outreach visits - Conduct ongoing training - Distribute educational materials - Facilitation - Organise clinician implementation team meetings - Promote adaptability - Promote network weaving | **Active dissemination**   - Conduct educational meetings - Distribute educational materials - Identify and prepare champions - Promote network weaving - Facilitation - Audit and provide feedback | **Evidence-based Group educational outreach session**   - Conduct educational outreach visits - Distribute educational materials | **Academic detailing**   - Conduct educational meetings - Conduct educational outreach visits - Distribute educational materials - Inform local opinion leaders - Alter incentive/allowance structures - Use mass media | **Promotional activity undertaken by pharmaceutical company**   - Conduct educational outreach visits - Use mass media |
| **Tools/ materials** | The intervention group received EBDI using MI and tailored to GPs’ thoughts on prompt and pragmatic benefit. | <https://asthma.atriumhealth.org/ToolKit>  The SDM Toolkit used in this project includes (1) a tool to assess  baseline asthma control, (2) a guide for eliciting the patient’s goals and priorities around medication options, (3) asthma educational materials, and (4) a tool to guide the negotiation process to jointly develop a treatment regimen that accommodates the patient’s goals and preferences. At the conclusion, an asthma action plan is provided. | Workshops and information materials, benchmarking data and email and telephone support | The comparative  information was summarised in a printed brochure, similar to  those given out by pharmaceutical companies  and was distributed during the visit.  During the session, oral and visual information was presented about the effectiveness and safety of the NSAIDs reviewed. | At these encounters, the investigator-educators selected from a centrally developed portfolio of training slides. Individualized communication with community physicians was encouraged, including specialized materials such as newsletters, pocket cards summarizing BP control messages, JNC7 reference cards, and other items relevant to CVD risk reduction. Press kits also were provided to investigator-educators to use with community media. | Promotional visits (detailing) & advertisements. |
| **Procedures** | All GPs present at the 66 participating PHCCs,  were presented with the new guidelines during a two  hour session. Information was tailored based on GPs thoughts & beliefs. | The facilitator-led method involved a participatory approach to the implementation of SDM to engage each practice. Each week over a period of 12 weeks, a trained facilitator from the research team held hour-long meetings at the practice. A core team  was invited to each  meeting with a goal to tailoring the adoption of the SDM intervention to the culture of the practice. The research team practice facilitator led the core team at the practice through a new training topic each week.  The facilitator also made themselves available for extra training and consultation throughout the study period.  Additionally, team members from the practices were invited to participate in a monthly call with the practice facilitators and PBRN researchers. | Report and recommendations sent. Workshop on organisational and behaviour change for regional champions. Workshop for unit staff on the research evidence, benchmarking, introduction to tools & practice change.  Benchmarking data then sent to sites, with support by phone and email. | The experimental intervention consisted of holding a group  educational outreach session with the GPs of each of the primary care teams. During the session, oral and visual information was presented about the effectiveness and safety of the NSAIDs reviewed. | Each investigator-educator’s goal was to make 12 presentations over a 12-month period to small, selected groups of key practitioners who prescribe medications for hypertension. | N/I |
| **Co-interventions** | Evidence-based drug information | Concurrent to this intervention, CCNC led a state-wide asthma intervention in all practices with Medicaid patients. Overlap between interventions occurred as the CCNC workgroup incorporated some asthma education materials from the SDM toolkit. CCNC practices also had access to a  facilitator to help improve management of asthma. | N/A | N/A | Several secondary project strategies for dissemination included reaching health care providers through professional associations and contacting formularies to encourage antihypertensive prescription according to JNC7 guidelines. Thus, the Dissemination Project had both local and national components aimed at increasing thiazide-type diuretic prescribing. | N/I |
| **Mode of delivery** | Face-to-face, group | Face-to-face with core team | Report and slide-set, Face-to-face group workshops and individual telephone and email contact. | Face-to-face group meeting | Face-to-face presentations | Face-to-face and via advertisements |
| **Who delivered the intervention?** | Medical information officers. (GPs and pharmacists). | Practice facilitators (PFs) (research nurses). The PFs were all registered nurses with bachelor’s degrees and had over 100 years of combined nursing experience. The PBRN PFs all had previous experience in research as well as health coaching and/or  practice facilitation with additional certifications including Certified Practice Facilitator (CPF), Certified Clinical Research Professional (CCRP), and Certified in User Experience (UXC). | CEMACH (research project) British Association of Perinatal Medicine. The attendees at Workshop 1 were supervised by trainers with expertise in organisational change. At Workshop 2, presentations were given by national clinical leaders. | The visits were led by the primary care pharmacist for the area of Teruel, who had been previously trained in the principles of evidence based medicine (EBM) and the basic tools for the dissemination of this information. | Investigator-educators, mainly clinical investigators from ALLHAT, were chosen based on their ability to recruit and retain ALLHAT participants plus the input of the ALLHAT leadership, especially the regional coordinators. Investigator-educators were recruited to cover most of the United States and to represent its demographic and geographic diversity. They were trained centrally to present ALLHAT results and the JNC7 guidelines as the foundation for achieving blood pressure (BP) control. | Pharmaceutical company reps |
| **Where was intervention provided?** | Primary healthcare centres | At participating primary care clinics in North Carolina, USA. | N/I | The study was conducted in the primary health care network  of the Instituto National de la Salud (National Institute of  Healthcare) in Teruel, a rural province in Aragon, Spain. The visits were held in each primary care centre. | N/I | N/I |
| **When and how often or much of the intervention was provided?** | One-off | 1 hour a week for 12 weeks.  After 1 year, the PF revisited their facilitator-led practices and met again with their core teams for “refresher” training sessions, aiming to promote sustainability of the intervention. The refresher sessions were 1 h-long meetings.  Throughout the 3-year project, the PFs were available to their facilitator-led practices for additional training or consultation as requested. Altogether the PFs spent a  minimum of 13 h on site at each practice,  plus an additional 1–4 h per week on average in the 18 months post rollout responding to various questions and concerns. The total dosage of PF support ranged from approximately 100–400 h. | 2 workshops (regional champions exposed to 2, others in active arm 1 or none). | One visit per primary care centre. | Low-level efforts occurred in 82 counties (target population, 12.2 million) with effort rates ranging from 0.3 to 15.9 clinician contacts per 100000 population 50 years or older (mean effort rate, 5.5).  Moderate-level efforts occurred in 79 counties (target population, 13.1 million) with effort rates ranging from 16 to 48 (mean effort rate, 30). High-level efforts occurred in 207 counties (target population, 10.2 million) with effort rates ranging from 50  to 5546 (mean effort rate, 136). | N/I |
| **Was the intervention tailored?** | Yes | Yes. This approach to dissemination allows clinics some freedom to tailor the Asthma Shared Decision Making (SDM) Toolkit and training process for their specific environment and patient population while maintaining fidelity of certain key elements that are felt to be essential for success. The PF assisted the core team in adapting the toolkit from the previous pilot study into a version that suited their practice’s specific needs. | Slideset was tailored for the specific audience, but not clear how. | No | Yes. Individualized communication with community physicians was encouraged, including specialized materials such as newsletters, pocket cards summarizing BP control messages, JNC7 reference cards, and other items relevant to CVD risk reduction. | N/I |
| **Was the approach modified or adapted?** | N/I | N/I | No | N/I | N/I | N/I |
| **How well was the approach delivered?** | N/I | All 10 facilitator-led practices remained engaged with their PFs, receiving at least 100 h of PF support in the 18 months post rollout, with 8 out of the 10 practices able to incorporate and sustain SDM visits or clinics. Of the 2 practices not able to fully implement, 1 practice experienced a 75% staffing turnover within the first year of the project. The other practice struggled with provider buy-in secondary to lack of leadership support in prioritizing SDM | N/I | Participation in the group sessions was  81.2% of the doctors in the experimental group. | N/I | N/I |

1. Skoglund I, Bjorkelund C, Petzold M, Gunnarsson R, Moller M. A randomized controlled trial comparing two ways of providing evidence-based drug information to GPs. Scandinavian Journal of Primary Health Care. 2013;31(2):67-72.

2. Ludden T, Shade L, Reeves K, Welch M, Taylor YJ, Mohanan S, et al. Asthma dissemination around patient-centered treatments in North Carolina (ADAPT-NC): a cluster randomized control trial evaluating dissemination of an evidence-based shared decision-making intervention for asthma management. J Asthma. 2019;56(10):1087-98.

3. Acolet D, Allen E, Houston R, Wilkinson AR, Costeloe K, Elbourne D. Improvement in neonatal intensive care unit care: A cluster randomised controlled trial of active dissemination of information. Archives of Disease in Childhood: Fetal and Neonatal Edition. 2011;96(6):F434-F9.

4. Bernal-Delgado E, Galeote-Mayor M, Pradas-Arnal F, Peiro-Moreno S. Evidence based educational outreach visits: effects on prescriptions of non-steroidal anti-inflammatory drugs. J Epidemiol Community Health. 2002;56(9):653-8.

5. Stafford RS, Bartholomew LK, Cushman WC, Cutler JA, Davis BR, Dawson G, et al. Impact of the ALLHAT/JNC7 dissemination project on thiazide-type diuretic use. Archives of Internal Medicine. 2010;170(10):851-8.

6. Majumdar SR, McAlister FA, Soumerai SB. Synergy between publication and promotion: comparing adoption of new evidence in Canada and the United States. Am J Med. 2003;115(6):467-72.

7. Powell BJ, Waltz TJ, Chinman MJ, Damschroder LJ, Smith JL, Matthieu MM, et al. A refined compilation of implementation strategies: results from the Expert Recommendations for Implementing Change (ERIC) project. Implementation Science. 2015;10(1):21.
