# Supplementary material for: The effectiveness of interventions to disseminate the results of non-commercial randomised clinical trials to healthcare professionals: a systematic review
Source: Implement Sci. 2024 Feb 1;19:8. doi: 10.1186/s13012-023-01332-w (PMC10835915; doi:10.1186/s13012-023-01332-w)
Supplement: Supplementary file 5 — Additional file 5: Table A5.1. GRADE rating of certainty of evidence for outreach interventions. This table shows the GRADE ratings of the included studies for outreach interventions. [file 13012_2023_1332_MOESM5_ESM.docx]

# Additional File 5: GRADE rating of certainty of evidence for outreach interventions

**Table A5.1 GRADE rating of certainty of evidence for outreach interventions**

| **GRADE domain** | **Judgement** | **Concerns about certainty domains** |
| --- | --- | --- |
| **Impact on practice** | | |
| Risk of bias | Two of the RCTs contributing to this outcome were judged to be at low risk of bias (one with 991 participants, and the other cRCT with 30 clusters and outcomes from 1228 patients), and there were some risk of bias concerns about the two other RCTs (180 clusters and 24 clusters) – in one case due to missing outcome data and discrepancies in the number of Units reported as randomised to intervention/control in protocol vs results paper, and in the other case due to lack of information about missing data and deviations from intended interventions. There were also some risk of bias concerns around one of the observational studies, and serious concerns about the other observational study (both observational studies were large). Using the approach recommended in the GRADE Handbook (1) we have been conservative in the judgement of rating down, and not rated down as we are not confident that there is substantial risk of bias across most of the available evidence, given the presence of two large, high quality studies. | Not serious |
| Indirectness | The population, interventions and comparators in most of the studies provide direct evidence to the question, with the exception being the Majumdar study, which provides indirect evidence (as this study uses a proxy measure (spend) for intensiveness of the intervention). There was variation in the outcome measures used, with four studies looking at changes in prescription practice, and two studies looking at other practice outcomes. | Not serious |
| Imprecision | The included studies cover a large number of participants/events and/or clusters, so this is not cause for concern over imprecision. | Not suspected |
| Inconsistency | One study (Skoglund 2013) at low risk of bias reported inconsistent effects, showing benefit at one timepoint and not another. All the other studies were consistent in reporting benefit across practice outcomes they measured, with the sign test direction of effect p-value=0.031. The magnitude of effect was less consistent, ranging from 4.2% to 37% absolute differences. | Downgraded by one level |
| Publication bias | We carried out a comprehensive search for studies, and there are several large studies, including one with inconsistent outcomes in this domain. | Not suspected. |
| Large effects | N/A | Not upgraded. |
| Dose response | One study reported a dose response gradient, but this was the only study to look at this, and was an observational study with some risk of bias concerns. | Not upgraded. |
| Opposing plausible residual bias and confounding | N/A | Not upgraded. |
| **Impact on policy** | | |
| Risk of bias | Only one study, which was a cRCT with some risk of bias concerns (due to missing outcome data and discrepancies in the number of Units reported as randomised to intervention/control in protocol vs results paper), contributes to this outcome. I have therefore downgraded the evidence by one level for this GRADE domain. | Downgraded by one level |
| Indirectness | The population, intervention, comparator and outcomes in the included study are directly relevant for the question of this review. | Not suspected |
| Imprecision | As each cluster only has one policy on each topic, the study has limited power to detect a difference in proportion of clusters with the relevant policy, especially as there was little scope for improvement as most units already had the desired policy. However, the confidence intervals are close to the point estimates. | Downgraded by one level |
| Inconsistency | Not applicable, as only one study contributes to this outcome domain | Not suspected |
| Publication bias | We carried out a comprehensive search for studies, but only found one study reporting the impact on policy of outreach interventions. | Not suspected |
| Large effects | Not applicable | Not upgraded |
| Dose response | Not applicable | Not upgraded |
| Opposing plausible residual bias and confounding | There was some baseline imbalance between the control and intervention arms in terms of proportion of units with the policies of interest, with a higher proportion of control arm units having the desired policies. | Upgraded by one level |
| **Impact on health** | | |
| Risk of bias | Two studies contribute to this outcome domain, one judged to be at low risk of bias, and the other having some risk of bias concerns (due to missing outcome data and discrepancies in the number of Units reported as randomised to intervention/control in protocol vs results paper). The study at some risk of bias randomised more clusters than the study with low risk of bias (180 vs 30), however, the study at low risk of bias had health outcomes from more individuals than the study with some risk of bias concerns (6,274 vs 355). I have therefore not downgraded based on risk of bias as the high quality study contributes much more information. | Not serious. |
| Indirectness | The population, intervention, comparator and outcomes in the included study are directly relevant for the question of this review. | Not suspected |
| Imprecision | The included studies cover a large number of participants/events and/or clusters, so this is not cause for concern over imprecision. | Not suspected |
| Inconsistency | Both studies showed benefit of outreach interventions in this outcome domain. The size of the absolute difference found in Ludden 2018 ranged from +0.7% (not statistically significant detriment) to -3.5% (statistically significant benefit), while Acolet found a 0.3⁰C (statistically significant) improvement in temperature. | Not serious |
| Publication bias | We carried out a comprehensive search for studies. | Not suspected |
| Large effects | Not applicable | Not upgraded |
| Dose response | Not applicable | Not upgraded |
| Opposing plausible residual bias and confounding | Not applicable | Not upgraded |
| **Impact on outcomes** | | |
| Risk of bias | Only one study, using an observational design, contributes evidence on ‘outcomes’. There are some risk of bias concerns around selection bias, and lack on information on response rate, and statistical methods. | Downgraded by one level |
| Indirectness | The population, intervention, comparator and outcomes in the included study are directly relevant for the question of this review. | Not suspected |
| Imprecision | Data were available from 2640 respondents. All outcomes reported showed statistically significant benefits (inconsistent with there being both an appreciable benefit and appreciable harm). | Not suspected |
| Inconsistency | The effects reported on outcomes range from +0.28 to +0.47 on a 5-point likert-type scale. The p-values were all ≤0.05. | Not suspected |
| Publication bias | We carried out a comprehensive search for studies, but only found one study reporting the impact on outcomes of outreach interventions. | Not suspected |
| Large effects | Not applicable | Not upgraded |
| Dose response | Not applicable | Not upgraded |
| Opposing plausible residual bias and confounding | Not applicable | Not upgraded |
| **Impact on out-takes** | | |
| Risk of bias | Only one study, using an observational design, contributes evidence on ‘outcomes’. There are some risk of bias concerns around selection bias, and lack on information on response rate, and statistical methods. | Downgraded by one level |
| Indirectness | The population, intervention, comparator and outcomes in the included study are directly relevant for the question of this review. | Not suspected |
| Imprecision | Data were available from 2640 respondents. All outcomes reported showed statistically significant benefits (inconsistent with there being both an appreciable benefit and appreciable harm). | Not suspected |
| Inconsistency | The effects reported on outcomes range from +0.12 to +0.49 on a 5-point likert-type scale. The p-values were all ≤0.05. | Not suspected |
| Publication bias | We carried out a comprehensive search for studies, but only found one study reporting the impact on out-takes of outreach interventions. | Not suspected |
| Large effects | Not applicable | Not upgraded |
| Dose response | Not applicable | Not upgraded |
| Opposing plausible residual bias and confounding | Not applicable | Not upgraded |

# References

1. Schunemann HJ, Brozek J, Gyatt G, A O. Quality of Evidence: GRADE Working Group; 2013 [Available from: <https://gdt.gradepro.org/app/handbook/handbook.html#h.svwngs6pm0f2>.
