# Supplementary material for: The effectiveness of interventions to disseminate the results of non-commercial randomised clinical trials to healthcare professionals: a systematic review
Source: Implement Sci. 2024 Feb 1;19:8. doi: 10.1186/s13012-023-01332-w (PMC10835915; doi:10.1186/s13012-023-01332-w)
Supplement: Supplementary file 6 — Additional file 6: Table A6.1. Summary of results of studies assessing the effectiveness of outreach interventions. This table summarises the results of included studies that assessed outreach interventions. [file 13012_2023_1332_MOESM6_ESM.docx]

# Additional File 6: Results of studies assessing the effectiveness of outreach interventions

**Table A6.1 Results of studies assessing the effectiveness of outreach interventions** (ordered by study design and risk of bias)

| **Study ID** | **Impact** | | **Narrative summary of results** |
| --- | --- | --- | --- |
|  | **Outcome measure** | **Results** |  |
| Skoglund 2013 (1) | Change in the proportion of ACE inhibitor prescriptions relative to the sum of ACE inhibitors and angiotensin II receptor blockers (intervention aimed to increase this proportion) | 0-3 months after intervention: Mean +0.14 (0.41SD) in intervention group  Mean +0.11 (0.49SD) in control group  4-6 months after intervention  Mean +0.11 (0.47SD) in intervention group  Mean +0.14 (0.48SD) in control group | Motivational interviewing added to Evidence Based Drug Information did not lead to change in prescription practices compared to Evidence Based Drug Information alone. |
| Ludden 2018 (2) | Patient perception of shared decision making (the provider and I participated equally in making the decision) | FLOW group: 528/705 (75%)  Traditional dissemination group: 347/523 (66%) | The FLOW dissemination approach did lead to more patients reporting that they participated equally in making the decision compared to traditional dissemination. However, this did not translate into significant improvements in the health outcomes measured. |
|  | Change in asthma emergency department visits | FLOW group: -1.3%  Traditional dissemination group: -1.6%  Control group: 1.1% |  |
|  | Change in asthma hospitalisation rate | FLOW group: 0.1%  Traditional dissemination group: -0.2%  Control group: -0.6% |  |
|  | Patients with oral steroid prescriptions | FLOW group: -5.8%  Traditional dissemination group: -5.1%  Control group: -3.6% |  |
|  | Patients with one or more exacerbation | FLOW group: -7.3%  Traditional dissemination group: -3.9%  Control group: -3.8% |  |
| Acolet 2011 (3) | Proportion of policies stating all intubated babies (<27 weeks) should receive surfactant within an hour of birth (as early as compatible with safety). | Not reported | The intervention does not seem to have had an effect on the policy outcomes, but does seem to have improved the practice outcomes |
|  | Proportion of units with a policy specifying which paediatric staff  should be present at an extremely preterm birth | Intervention: 55/61 Units (90%)  Control: 67/68 Units (99%) |  |
|  | Proportion of births <27 weeks gestational age, where the following were present before the baby is delivered: a consultant paediatrician or a middle grade practitioner and a Senior House Officer or an Advanced Neonatal Nurse Practitioner. | Intervention: 126/169 (68%)  Control: 97/186 (57%) |  |
|  | Proportion of intubated babies (<27 weeks) who receive surfactant within an hour of birth | Intervention: 141/169 (83%)  Control: 96/186 (52%) |  |
|  | Proportion of units with a strategy for hypothermia prevention | Intervention: 61/62 Units (98%)  Control: 67/68 (99%) |  |
|  | Proportion of babies whose Core temperature on admission to NICU is ≥ 36°C | Not reported. However, mean temperature on admission to neonatal unit was 36.5 (0.08) for intervention, and 36.2 (0.08) for the control |  |
|  | Proportion of babies whose trunk was delivered into a plastic bag to avoid hypothermia | Intervention: 141/168 (79%)  Control: 93/186 (62%) |  |
| Bernal-Delgado 2002 (4) | Proportional changes in number of diclofenac prescriptions, comparing six months after the intervention with six months before (intervention aimed to increase use) | Intervention group: -9% (-17 to -1)  Placebo group: -12% (-20 to -3)  Control group: -17% (-26 to -7) | There was a greater reduction in the drugs not recommended in the intervention group than the other two groups. Use of the recommended drug fell less in the intervention group than the other two groups. This suggests the intervention did have an effect on prescribing practice. |
|  | Proportional changes in number of meloxicam prescriptions (not recommended in intervention) | Intervention group: -26% (-38 to -13)  Placebo group: -13% (-22 to -4)  Control group: -1% (-4 to -2) |  |
|  | Proportional changes in number of tenoxicam prescriptions (not recommended in intervention) | Intervention group: -23% (-38 to -13)  Placebo group: -10% (-18 to -2)  Control group: 14% (5 to 23) |  |
| Stafford 2010 and Bartholomew 2009 (5, 6) | Among drug-treated visits for hypertension, the change in percentage of patients prescribed Thiazides | High effort areas: 8.6  Moderate effort areas: 1.9  Low effort areas: -1.6  No effort areas: 2.3 | The intervention consistently improved reported self-efficacy, expectations of benefit from changing prescribing practices, and intention to change practice.  The data on impact suggests that areas exposed to the highest ‘dose’ of the intervention saw the greatest changes in prescription practice. Those areas with low exposure to the intervention were not statistically different to areas receiving no intervention. |
|  | The number of thiazide-type diuretic prescriptions dispensed by pharmacists per 1000 persons | High effort areas: 8.1% increase  Moderate effort areas: 10% increase  Low effort areas: 7.8%  No effort areas: 3.9% |  |
| Majumdar 2003 (7) | Monthly percentage changes in prescriptions dispensed for ramipril (intervention aimed to increase this) | Canada (intervention): 12% per month increase  USA (control): 5% per month increase | The study found a greater increase in the use of ramipril in Canada, where the drug was promoted via detailing by the pharmaceutical company, compared to the USA, where detailing did not take place.  The comparison study, where no detailing took place in either country, saw similar changes in prescriptions in both the USA and Canada.  This suggests that pharmaceutical company does lead to changes in practice over and above what would be expected from the ‘publication effect’. |
|  | Monthly percentage changes in prescriptions dispensed for all other ACE inhibitors | Canada (intervention): No change  USA (control): 0.9% per month increase |  |
|  | The monthly percentage changes in prescriptions dispensed for spironolactone (publication effect) | Canada: 2% per month increase  USA: 2% per month increase |  |
|  | Proportion of ACE inhibitor market accounted for by ramipril | Canada (intervention): increase from 6% pre to 30% post  USA (control): increase from 4% pre to 6% post |  |

# References

1. Skoglund I, Bjorkelund C, Petzold M, Gunnarsson R, Moller M. A randomized controlled trial comparing two ways of providing evidence-based drug information to GPs. Scandinavian Journal of Primary Health Care. 2013;31(2):67-72.

2. Ludden T, Shade L, Reeves K, Welch M, Taylor YJ, Mohanan S, et al. Asthma dissemination around patient-centered treatments in North Carolina (ADAPT-NC): a cluster randomized control trial evaluating dissemination of an evidence-based shared decision-making intervention for asthma management. J Asthma. 2019;56(10):1087-98.

3. Acolet D, Allen E, Houston R, Wilkinson AR, Costeloe K, Elbourne D. Improvement in neonatal intensive care unit care: A cluster randomised controlled trial of active dissemination of information. Archives of Disease in Childhood: Fetal and Neonatal Edition. 2011;96(6):F434-F9.

4. Bernal-Delgado E, Galeote-Mayor M, Pradas-Arnal F, Peiro-Moreno S. Evidence based educational outreach visits: effects on prescriptions of non-steroidal anti-inflammatory drugs. J Epidemiol Community Health. 2002;56(9):653-8.

5. Stafford RS, Bartholomew LK, Cushman WC, Cutler JA, Davis BR, Dawson G, et al. Impact of the ALLHAT/JNC7 dissemination project on thiazide-type diuretic use. Archives of Internal Medicine. 2010;170(10):851-8.

6. Bartholomew LK, Cushman WC, Cutler JA, Davis BR, Dawson G, Einhorn PT, et al. Getting clinical trial results into practice: design, implementation, and process evaluation of the ALLHAT Dissemination Project. Clin Trials. 2009;6(4):329-43.

7. Majumdar SR, McAlister FA, Soumerai SB. Synergy between publication and promotion: comparing adoption of new evidence in Canada and the United States. Am J Med. 2003;115(6):467-72.
