# Supplementary material for: The effectiveness of interventions to disseminate the results of non-commercial randomised clinical trials to healthcare professionals: a systematic review
Source: Implement Sci. 2024 Feb 1;19:8. doi: 10.1186/s13012-023-01332-w (PMC10835915; doi:10.1186/s13012-023-01332-w)
Supplement: Supplementary file 7 — Additional file 7: Table A7.1. Description of the systematic review summary format interventions. This table describes the interventions examined in the studies of systematic review summary formats. [file 13012_2023_1332_MOESM7_ESM.docx]

# Additional File 7: Description of the systematic review summary format interventions

All the interventions in this category were employing the implementation strategy of ‘develop educational materials’(1), as all the systematic review summary formats were designed to make it easier for stakeholders to learn about the intervention (improving their ability to use and apply the evidence).

**Table A7.1: Description of the systematic review summary format interventions**

| **Study** | **Opiyo 2013 (2)** | | | **Vandvik 2012 (3)** | | **Neumann 2018 (4)** | | **Rosenbaum 2010a (5)** | | **Gartlehner 2017 (6)** | |
| --- | --- | --- | --- | --- | --- | --- | --- | --- | --- | --- | --- |
| **Intervention name** | **Systematic review + summary-of-findings table** | **Graded-entry format** | **Systematic review alone** | **Table A** | **Table B** | **Recommendation with evidence summary** | **Evidence summary alone** | **Summary of Findings table (formatted)** | **Summary of Findings table (unformatted)** | **Fishbone diagram** | **Summary of Findings table** |
| **Tools/ materials** | Systematic review + summary-of-findings table | It started with a ‘front-end’ short  interpretation of the main findings and conclusions, drawn from evidence synthesis. These front-end concise summaries were followed by a locally prepared, short, contextually framed, narrative report in which the results of the systematic review (and other evidence  where relevant) were described and locally relevant factors that could influence the implementation of evidence-based guideline  recommendations (e.g. resource capacity) were highlighted. The  front-end summary and the narrative report were combined with the full systematic review  to make a three-component set branded pack ‘C’. | Systematic review alone | We used the currently available evidence profile format (also see in the Appendix on the  journal’s Web site at www.elsevier.com) as the starting point and performed some minor formatting changes in adherence with general design principles and results from the SoF-table studies | | Recommendations were presented as they appeared in the original publication, including the text of the recommendation, the rating of the certainty of the evidence, the strength of the recommendation, and any other remarks. The strength of the recommendation was labelled in the published guidelines as ‘‘strong’’ or ‘‘weak,’’ following GRADE nomenclature. Strong recommendations reflect treatment alternatives in which the benefits of the intervention clearly outweigh the harms and in which it is anticipated that all or almost all fully informed individuals would choose the treatment. Weak recommendations reflect a closer balance between benefits and harms with anticipated larger variation in treatment choices and courses of action, depending on values and preferences of patients or contextual factors.  Evidence summaries were presented in a tabular format following the recommendations of the Cochrane Collaboration and the GRADE working group for summary of findings tables. | Evidence summaries were presented in a tabular format  following the recommendations of the Cochrane Collaboration  and the GRADE working group for summary of findings tables. In addition, when the recommendation  was not presented, we developed a narrative summary of the same evidence to both to provide a similar amount of  information in both arms of the trial and to ensure that respondents with different preferences for presentation formats received the information in a manner with which they were comfortable with. | Summary of Findings table | Summary of Findings table with limited formatting, as would be possible in current Cochrane review software | The “head” represents the overall balance between benefits and harms of an intervention or of competing interventions.  The bones of the fish represent individual outcomes that are critical or important to balance the overall benefits  and harms of the interventions. The proximity of the bones to the head reflects the importance of outcomes  for decision making. GRADE, for example, recommends ranking the relative importance of outcomes when developing guidelines. Each bone (representing an outcome) can include additional explanatory factors such as a plain language summary of the effect, the number of  studies, the magnitude of treatment effects, or other factors that influence the certainty (strength) of a body of  evidence. | Summary of Findings table |
|  |  |  |  | Placement of additional information: rapid retrieval  Placement of overall quality of evidence: Under heading ‘Quality assessment’  Study event rates: not reported to reduce clutter  Absolute risk differences: reported to forgo need for calculation | Placement of additional information: Uncluttered table  Placement of overall quality of evidence: Under heading ‘Summary of Findings’  Study event rates: Reported (to be informative)  Absolute risk differences: not reported to avoid potential confusion |  |  |  |  |  |  |
| **Procedures** | The information packs were delivered to participants as pre-reading materials one month before the workshop. | | | Panelists were asked to do the following: (1) answer questions about their background and download and print the evidence profile to  which they were allocated, (2) review the evidence profile and answer questions about comprehension and accessibility, (3) record time before and after answering questions,  and (4) download and study the second evidence profile and report preferences for specific formatting alternatives and overall format of tables. | | Within each group, they provided respondents with an evidence summary for one scenario and with an evidence summary plus a recommendation for the other scenario. Participants were also randomized to the order in which they received the recommendation, that is, the first or second clinical scenario. | | The first trial took place during a plenary session at a workshop for newcomers to evidence-based practice. Participants were asked if they would help evaluate ways of making reviews more accessible.  Questionnaires with the interventions were distributed to participants. | | The participants were given a brief summary text that introduced the topic (preoperative anemia management) represented in the two displays. | |
| **Co-interventions** | Each participant received one pack of each type (topic of pack was randomised) | | | Other version of the table was looked at after they had completed the first set of questions. | | Each participant received an evidence summary alone for one scenario, and an evidence summary plus recommendation for the other scenario. | | Workshop / meeting | | N/A | |
| **Mode of delivery** | Materials were sent to participants one month before the workshop. | | | Online | | Paper surveys distributed at meetings | | Group – workshop/meeting | | Computer | |
| **Who delivered the intervention?** | N/I | | | Guideline development executive committee | | N/I | | N/I | | N/I | |
| **Where was intervention provided?** | Kenya | | | Online | | Grand rounds or clinical meetings | | Workshop for newcomers to evidence-based practice (RCT 1)  Meeting for members of Continental European Cochrane entities (RCT 2) | | University (Austria) | |
| **When and how often or much of the intervention was provided?** | Each participant received packs on three topics (one pack A, one B and one C). | | | One-off | | One-off | | One-off | | One-off within a class | |
| **Was the intervention tailored?** | No | | | No | | No | | No | | No | |
| **Was the approach modified or adapted?** | No | | | No | | No | | The format was modified based on the findings of RCT 1. | | N/I | |
| **How well was the approach delivered?** | N/I | | | N/I | | N/I | | N/I | | N/I | |

# References

1. Powell BJ, Waltz TJ, Chinman MJ, Damschroder LJ, Smith JL, Matthieu MM, et al. A refined compilation of implementation strategies: results from the Expert Recommendations for Implementing Change (ERIC) project. Implementation Science. 2015;10(1):21.

2. Opiyo N, Shepperd S, Musila N, Allen E, Nyamai R, Fretheim A, et al. Comparison of Alternative Evidence Summary and Presentation Formats in Clinical Guideline Development: A Mixed-Method Study. PLoS ONE. 2013;8 (1) (no pagination)(e55067).

3. Vandvik PO, Santesso N, Akl EA, You J, Mulla S, Spencer FA, et al. Formatting modifications in GRADE evidence profiles improved guideline panelists comprehension and accessibility to information. A randomized trial. J Clin Epidemiol. 2012;65(7):748-55.

4. Neumann I, Alonso-Coello P, Vandvik PO, Agoritsas T, Mas G, Akl EA, et al. Do clinicians want recommendations? A multicenter study comparing evidence summaries with and without GRADE recommendations. Journal of Clinical Epidemiology. 2018;99:33-40.

5. Rosenbaum SE, Glenton C, Oxman AD. Summary-of-findings tables in Cochrane reviews improved understanding and rapid retrieval of key information. J Clin Epidemiol. 2010;63(6):620-6.

6. Gartlehner G, Schultes MT, Titscher V, Morgan LC, Bobashev GV, Williams P, et al. User testing of an adaptation of fishbone diagrams to depict results of systematic reviews. BMC Med Res Methodol. 2017;17(1):169.
