# Supplementary material for: The effectiveness of interventions to disseminate the results of non-commercial randomised clinical trials to healthcare professionals: a systematic review
Source: Implement Sci. 2024 Feb 1;19:8. doi: 10.1186/s13012-023-01332-w (PMC10835915; doi:10.1186/s13012-023-01332-w)
Supplement: Supplementary file 8 — Additional file 8: Table A8.1. GRADE rating of certainty of evidence for systematic review summary formats. This table shows the GRADE ratings of the included studies for systematic review summary formats. [file 13012_2023_1332_MOESM8_ESM.docx]

# Additional File 8: GRADE rating of certainty of evidence on systematic review summary formats

**Table A8.1: GRADE rating of certainty of evidence on systematic review summary formats**

| **GRADE domain** | **Judgement** | **Concerns about certainty domains** |
| --- | --- | --- |
| **Impact on outcomes** | | |
| Risk of bias | Two of the included studies were at low risk of bias, and one at some risk of bias due to lack of information on randomisation, blinding and deviations from intended interventions, and proportions of missing data across intervention groups. Using the approach recommended in the GRADE Handbook [7] we have been conservative in the judgement of rating down, and not rated down as we are not confident that there is substantial risk of bias across most of the available evidence, given the presence of two high quality studies. | Not serious (not downgraded) |
| Indirectness | The population, intervention and outcome are directly relevant to the question of this review. | Not suspected |
| Imprecision | Two of the included studies had relatively small samples sizes, while one was moderately sized, giving a total of 661 participants across the three studies. | Not suspected |
| Inconsistency | There is substantial inconsistency in the direction and magnitude of effects between the three studies (and between different outcome measures in some of the studies). | Downgraded by one level |
| Publication bias | We carried out a comprehensive search for studies, and found three studies, reporting different directions of effects. | Not suspected |
| Large effects | Not applicable | Not upgraded |
| Dose response | Not applicable | Not upgraded |
| Opposing plausible residual bias and confounding | Not applicable | Not upgraded |
| **Impact on out-takes** | | |
| Risk of bias | Three of the included studies were at low risk of bias, and there were risk of bias concerns about two of the studies: one due to the possibility of cross contamination if participants could read their neighbour’s Summary of Findings table, and one due to lack of information on randomisation, blinding and deviations from intended interventions, and proportions of missing data across intervention groups. Excluding the two studies at some risk of bias would be unlikely to change our interpretation of the overall effect summary formats for systematic reviews | Not serious (not downgraded) |
| Indirectness | The population, intervention and outcome are directly relevant to the question of this review. | Not suspected |
| Imprecision | The studies included 831 participants, so imprecision is not suspected. | Not suspected |
| Inconsistency | Three of the included studies reported mixed effects across different ‘out-take’ measures, while two studies reported some overall benefit on ‘out-takes’. | Downgraded by one level |
| Publication bias | We carried out a comprehensive search for studies, and found five studies, reporting different directions of effects. | Not suspected |
| Large effects | Not applicable | Not upgraded |
| Dose response | Not applicable | Not upgraded |
| Opposing plausible residual bias and confounding | Not applicable | Not upgraded |
