# Supplementary material for: The effectiveness of interventions to disseminate the results of non-commercial randomised clinical trials to healthcare professionals: a systematic review
Source: Implement Sci. 2024 Feb 1;19:8. doi: 10.1186/s13012-023-01332-w (PMC10835915; doi:10.1186/s13012-023-01332-w)
Supplement: Supplementary file 9 — Additional file 9: Table A9.1. Summary of results of studies assessing the effectiveness of systematic review summary formats. This table summarises the results of included studies assessing the effectiveness of systematic review summary formats. [file 13012_2023_1332_MOESM9_ESM.docx]

# Additional File 9: Results of the studies assessing the effectiveness of systematic review summary formats

**Table A9.1 Results of the studies assessing the effectiveness of systematic review summary formats**

| **Study ID** | **Out-takes** | | **Outcomes** | | **Summary of results** |
| --- | --- | --- | --- | --- | --- |
|  | **Outcome measure** | **Results** | **Outcome measure** | **Results** |  |
| Opiyo 2010 (1) | Correct responses to key clinical questions relevant to the specific tracer topics. | 79/130 with Summary of findings table  80/129 with graded entry  91/130 for Systematic review alone |  |  | There were no significant differences between the pack types in the odds of correct responses to clinical questions.  The graded entry format was superior to the systematic review alone in terms of value and accessibility score. The majority of respondents strongly preferred the graded entry format to the systematic review alone. |
|  | Value and accessibility score | -0.14 mean difference with the Summary of Findings Table  0.49 mean difference with the graded entry format  (Compared to Systematic review alone) |  |  |  |
|  | Preferences | 53% strongly prefer narrative report to systematic review  40% strongly prefer summary of finding table to systematic review  38% strongly prefer narrative report to summary of finding table |  |  |  |
| Vandvik 2012 (2) | Comprehension: for the outcome mortality, why is the quality of evidence rated as moderate? | Table A: 97% correct (38/39)  Table B: 92% correct (35/38) | Preference for presentation of additional info in table cells rather than footnotes | Median: 2 (respondents preferred the information to be in the table (as in Table A)) | Table A performed better than Table B on two comprehension outcomes, but there was no significant difference in the other comprehension outcomes. Participants spent less time studying Table A. There were no significant differences in accessibility outcomes between the two table formats, nor was one format preferred overall compared to the other format. |
|  | Comprehension: What was the specific reason for rating down for imprecision? | Table A: 88% correct (35/40)  Table B: 86% correct (31/36) | Preference for presentation of study event rates | Median: 1 (preferred presentation of study event rates (as in Table B) rather than no study event rates) |  |
|  | Comprehension: For what period of time are the risk estimates calculated? | Table A: 58% correct (23/40)  Table B: 11% correct (4/36) | Preference for presentation of absolute risk differences | Median 2 (preference for risk differences (as in Table A) rather than absolute events) |  |
|  | Comprehension: What is the absolute effect on mortality if people take LMWH vs VKA for 6 months? | Table A: 80% correct (32/40)  Table B: 69% correct (19/35) | Preference for placement of column for overall quality of evidence | No significant preference found for placement of column for overall quality of evidence |  |
|  | Comprehension: What is the range in which that effect might lie? | Table A: 95% correct (38/40)  Table B: 54% correct (19/35) | Preference for overall table format (A vs B) | No significant preference found for overall table format |  |
|  | Comprehension: Consider a patient who has nonmetastatic cancer. Over 6 months, what is the patient’s risk of having recurrent symptomatic VTE while taking VKAs? | Table A: 83% correct (33/40)  Table B: 77% correct (27/35) |  |  |  |
|  | Accessibility | There was no significant interaction between clinical question and table format for any of the accessibility variables. |  |  |  |
|  | Time spent studying the evidence profile | Table A: 4 minutes median, IQR 2  Table B: 6 minutes median, IQR 4 |  |  |  |
| Neumann 2018 (3) | Clinicians’ preference for evidence summaries plus a recommendation vs. evidence summaries alone. | For the scenarios addressing strong recommendations, 189/219 (86%) participants preferred having recommendations in addition to evidence summaries (p<0.001).  On the scenarios related to weak recommendations, 201/248 (81%) preferred the addition of recommendation for the scenarios presented. | Would recommend oseltamivir for avian influenza | Recommendation + evidence summary: 94/119  Evidence summary alone: 60/112 | Clinicians expressed a strong preference for having a recommendation alongside the evidence summary, rather than the evidence summary alone. The presence of a recommendation did not improve knowledge about the subject, but did result in more clinicians reporting an appropriate intended course of action for the two scenarios with strong recommendations. |
|  | Clinicians’ preference for evidence summaries plus a recommendation vs evidence summaries alone in the context of typical clinical practice | For strong recommendations: 193/219 (88%) preferred having recommendations in the context of typical clinical practice  For weak recommendations: 215/248 (87%) preferred having recommendations for clinical practice | Would not recommend aspirin for patients with asymptomatic thrombophilia | Recommendation + evidence summary: 101/111  Evidence summary alone: 102/120 |  |
|  | Participants answering all questions about oseltamivir correctly | Recommendation + evidence summary: 45/115  Evidence summary alone: 43/109 |  |  |  |
|  | Participants answering all questions about aspirin correctly | Recommendation + evidence summary: 27/107  Evidence summary alone: 38/119 |  |  |  |
|  | Participants answering all questions about potassium intake correctly | Recommendation + evidence summary: 51/126  Evidence summary alone: 52/122 |  |  |  |
|  | Participants answering all questions about compression stockings correctly | Recommendation + evidence summary: 55/125  Evidence summary alone: 69/121 |  |  |  |
|  | Benefits of oral oseltamivir >>harms | Recommendation + evidence summary: 34/118  Evidence summary alone: 11/119 |  |  |  |
|  | Harms of aspirin for asymptomatic thrombophilia >> benefits | Recommendation + evidence summary: 44/109  Evidence summary alone: 41/118 |  |  |  |
|  | Daily potassium intake of more than 3.5g benefits >> harms | Recommendation + evidence summary: 9/125  Evidence summary alone: 3/120 |  |  |  |
|  | Compression stockings benefits >> harms | Recommendation + evidence summary: 61/125  Evidence summary alone: 69/124 |  |  |  |
| Rosenbaum 2010a (4) | Agreeing review authors have indicated what they believe are the most important outcomes | 81% with the Summary of Findings table vs 80% without |  |  | The summary of findings table improved understanding of the results, and sped up retrieval of information from a systematic review. |
|  | Agreeing it was easy to find this information (about which outcomes are important) | 72% with the Summary of Findings table vs 60% without |  |  |  |
|  | Agreeing it was easy to find results for important outcomes | 68% with the Summary of Findings table vs 40% without |  |  |  |
|  | Agreeing it was easy to find quality of the evidence for important outcomes | 43% with the Summary of Findings table vs 24% without |  |  |  |
|  | Agreeing it was easy to understand the main findings | 68% with the Summary of Findings table vs 56% without |  |  |  |
|  | Agreeing main findings are presented in a such a way that they would be helpful to me in making a decision | 60% with the Summary of Findings table vs 52% without |  |  |  |
|  | Agreeing findings are presented in an accessible way | 41% with the Summary of Findings table vs 17% without |  |  |  |
|  | % correct answer to: What is the risk of symptomless DVT after a long flight for people at low risk who do not wear compression stockings? | 93% with the Summary of Findings table vs 44% without |  |  |  |
|  | % correct answer to: What would be the risk if they wore stockings? | 87% with the Summary of Findings table vs 11% without |  |  |  |
|  | % correct answer to: How much confidence do the review authors have in the estimated effect of stockings on the risk of symptomless DVT? | 87% with the Summary of Findings table vs 67% without |  |  |  |
|  | % correct answer to: What are the most important outcomes? | 33% with the Summary of Findings table vs 53% without |  |  |  |
|  | Minutes spent finding answer to What is the risk of symptomless deep vein thrombosis (DVT) after a long flight for people at low risk who do not wear compression stockings? | 1.5 with the Summary of Findings table vs 4 without |  |  |  |
|  | Minutes spent finding answer to What would be the risk if they wore stockings? | 1.3 with the Summary of Findings table vs 2.8 without |  |  |  |
|  | Minutes spent finding answer to How much confidence do the review authors have in the estimated effect of stockings on the risk of symptomless DVT? | 2.1 with the Summary of Findings table vs 1.5 without |  |  |  |
|  | Minutes spent finding answer to What are the most important outcomes? | 2.0 with the Summary of Findings table vs 1.9 without |  |  |  |
|  | % who would not have needed more time to find this information | 79% with the Summary of Findings table vs 64% without |  |  |  |
| Gartlehner 2017 (5) | Correct overall conclusion | Fishbone diagram: 68.4%  Summary of Findings table: 71.8% | Strongly agree/agree/slightly agree they liked the diagram/table | Fishbone diagram: 44.8%  Summary of Findings table: 71.8% | Participants in the Summary of Findings table group were more likely to answer comprehension questions correctly than those who were in the Fishbone diagram group. Participants preferred the Summary of Findings table to the Fishbone diagram, finding it easier to use and understand, and less confusing. However, of those who correctly answered questions, those in the Fishbone diagram group answered correctly faster than those in the Summary of Findings table group. |
|  | Time to get correct conclusion (among those who answered correctly) | Fishbone diagram: 154.5 seconds  Summary of Findings table: 180.8 seconds | Would recommend summary format to a colleague | Fishbone diagram: 11.7%  Summary of Findings Table: 53.2% |  |
|  | Answered 3 questions correctly that required finding and interpreting facts | Fishbone diagram: 52.6%  Summary of Findings table: 51.3% | Would recommend summary format to a systematic reviewer | Fishbone diagram: 7.8%  Summary of Findings table: 74% |  |
|  | Time to get 3 correct answers (among those who got 3 correct answers) | Fishbone diagram: 123.2 seconds  Summary of Findings table: 149.3 seconds | Would recommend summary format to a patient | Fishbone diagram: 31.2%  Summary of Findings Table: 23.4% |  |
|  | Strongly agree/agree/slightly agree it was hard to find the information I was interested in | Fishbone diagram: 65.8%  Summary of Findings table: 45% |  |  |  |
|  | Strongly agree/agree/slightly agree the information in the diagram/table was confusing | Fishbone diagram: 63.2%  Summary of Findings table: 35.9% |  |  |  |
|  | Easier to use | 19.83 (13.5, 26.16) (above 0 favours Summary of Findings table) |  |  |  |
|  | Easier to understand | 15.14 (8.09, 22.19) (above 0 favours Summary of Findings table) |  |  |  |
|  | Preferable | 17.38 (10.95, 23.8) (above 0 favours Summary of Findings table) |  |  |  |
|  | Less confusing | 19.82 (13.38, 26.25) (above 0 favours Summary of Findings table) |  |  |  |
|  | Better designed | -1.55 (-8.36, 5.27) (above 0 favours Summary of Findings table) |  |  |  |

# References

1. Opiyo N, Shepperd S, Musila N, Allen E, Nyamai R, Fretheim A, et al. Comparison of Alternative Evidence Summary and Presentation Formats in Clinical Guideline Development: A Mixed-Method Study. PLoS ONE. 2013;8 (1) (no pagination)(e55067).

2. Vandvik PO, Santesso N, Akl EA, You J, Mulla S, Spencer FA, et al. Formatting modifications in GRADE evidence profiles improved guideline panelists comprehension and accessibility to information. A randomized trial. J Clin Epidemiol. 2012;65(7):748-55.

3. Neumann I, Alonso-Coello P, Vandvik PO, Agoritsas T, Mas G, Akl EA, et al. Do clinicians want recommendations? A multicenter study comparing evidence summaries with and without GRADE recommendations. Journal of Clinical Epidemiology. 2018;99:33-40.

4. Rosenbaum SE, Glenton C, Oxman AD. Summary-of-findings tables in Cochrane reviews improved understanding and rapid retrieval of key information. J Clin Epidemiol. 2010;63(6):620-6.

5. Gartlehner G, Schultes MT, Titscher V, Morgan LC, Bobashev GV, Williams P, et al. User testing of an adaptation of fishbone diagrams to depict results of systematic reviews. BMC Med Res Methodol. 2017;17(1):169.
